# The Laboratory-Based Intermountain Validated Exacerbation (LIVE) Score Identifies Chronic Obstructive Pulmonary Disease Patients at High Mortality Risk

**DOI:** 10.3389/fmed.2018.00173

**Published:** 2018-06-11

**Authors:** Denitza P. Blagev, Dave S. Collingridge, Susan Rea, Benjamin D. Horne, Valerie G. Press, Matthew M. Churpek, Kyle A. Carey, Richard A. Mularski, Siyang Zeng, Mehrdad Arjomandi

**Affiliations:** ^1^Division of Pulmonary and Critical Care Medicine, Department of Medicine, Intermountain Medical Center, Murray, UT, United States; ^2^Division of Respiratory, Critical Care, and Sleep Medicine, Department of Medicine, University of Utah School of Medicine, Salt Lake City, UT, United States; ^3^Office of Research, Intermountain Healthcare, Salt Lake City, UT, United States; ^4^Homer Warner Center for Informatics Research, Murray, UT, United States; ^5^Intermountain Medical Center, Intermountain Heart Institute, Murray, UT, United States; ^6^Department of Biomedical Informatics, University of Utah, Salt Lake City, UT, United States; ^7^Section of General Internal Medicine, Department of Medicine, University of Chicago Medicine, Chicago, IL, United States; ^8^Section of Pulmonary and Critical Care Medicine, Department of Medicine, University of Chicago Medicine, Chicago, IL, United States; ^9^Kaiser Permanente Center for Health Research—Northwest, Portland, OR, United States; ^10^Division of Pulmonary and Critical Care Medicine, Oregon Health & Science University, Portland, OR, United States; ^11^Division of Pulmonary and Critical Care Medicine, San Francisco Veterans Affairs Medical Center, San Francisco, CA, United States; ^12^Division of Pulmonary and Critical Care Medicine, University of California, San Francisco, San Francisco, CA, United States

**Keywords:** COPD, cluster analysis, comorbidity, risk stratification, informatics, LIVE Score

## Abstract

**Background:** Identifying COPD patients at high risk for mortality or healthcare utilization remains a challenge. A robust system for identifying high-risk COPD patients using Electronic Health Record (EHR) data would empower targeting interventions aimed at ensuring guideline compliance and multimorbidity management. The purpose of this study was to empirically derive, validate, and characterize subgroups of COPD patients based on routinely collected clinical data widely available within the EHR.

**Methods:** Cluster analysis was used in 5,006 patients with COPD at Intermountain to identify clusters based on a large collection of clinical variables. Recursive Partitioning (RP) was then used to determine a preferred tree that assigned patients to clusters based on a parsimonious variable subset. The mortality, COPD exacerbations, and comorbidity profile of the identified groups were examined. The findings were validated in an independent Intermountain cohort and in external cohorts from the United States Veterans Affairs (VA) and University of Chicago Medicine systems.

**Measurements and Main Results:** The RP algorithm identified five LIVE Scores based on laboratory values: albumin, creatinine, chloride, potassium, and hemoglobin. The groups were characterized by increasing risk of mortality. The lowest risk, LIVE Score 5 had 8% 4-year mortality vs. 56% in the highest risk LIVE Score 1 (*p* < 0.001). These findings were validated in the VA cohort (*n* = 83,134), an expanded Intermountain cohort (*n* = 48,871) and in the University of Chicago system (*n* = 3,236). Higher mortality groups also had higher COPD exacerbation rates and comorbidity rates.

**Conclusions:** In large clinical datasets across different organizations, the LIVE Score utilizes existing laboratory data for COPD patients, and may be used to stratify risk for mortality and COPD exacerbations.

## Introduction

Chronic obstructive pulmonary disease (COPD) is a disease of increasing prevalence and mortality worldwide (1, 2). While pulmonary function tests (PFT) are the cornerstone of diagnosis and treatment of COPD, functional impairment, disability, and overall mortality have been inadequately predicted by the forced expiratory volume in 1 second (FEV_1_) alone (3–8). COPD exacerbation frequency and mortality in COPD patients are driven not only by the severity of COPD, but also by the type, number, and severity of associated comorbidities (5, 9–19). Several risk stratification tools have been developed, which predict mortality in COPD patients (20–22). However, none of the existing risk stratification tools allow for identification of high-risk COPD patients on a system level.

Cluster analysis techniques have been used in empirically identifying groups of patients diagnosed under a common disease umbrella in other fields. For example, cluster analysis of patients with severe asthma identified five subgroups of patients with asthma who have unique characteristics and profiles (23). More recently, cluster analysis has been used to identify subgroups of patients with diabetes (24). While prior risk scores in COPD have used PFT and dyspnea scores to identify subgroups of COPD patients (20), those data are not routinely available to be queried in most current Electronic Health Records (EHRs), and thus have limited utility when designing interventions to improve COPD care within a healthcare system.

While PFT data and dyspnea scores are not routinely available to be queried in an EHR, many other clinically collected variables are accessible. For example, in cardiology, increased red cell distribution width (RDW) is associated with increased cardiovascular mortality (25–29). Although RDW is a marker for disease, rather than a primary driver, the reliance on laboratory values to derive a risk score allows for the identification of high-risk patients in real time during a healthcare encounter (27, 30). This ability to identify patients at risk for cardiovascular mortality in real time, has facilitated the development of focused interventions with increased resources and coordination of care for high-risk patients. A similar approach has been shown to be effective in improving outcomes for floor patients at risk of developing sepsis (31). Thus, risk scores have been most useful in improving care for patients when individual patient risk can be assessed automatically and an alert surfaced to clinicians for additional care only in those with high-risk.

Despite the advances in risk scores, finding a COPD related risk stratification score that allows system wide identification of patients who may benefit from targeted interventions remains elusive. Given the large number of variables routinely collected as part of clinical care, we wanted to determine whether clustering COPD patients would identify different subgroups that may have differential mortality, exacerbation frequency, or comorbidity rates.

## Methods

The Institutional Review Boards at Intermountain Healthcare, the University of California San Francisco, the San Francisco Veterans Affairs Medical Center Research and Development Committee, and the University of Chicago Medicine approved this retrospective data-only study and waived individual informed consent.

### Dataset

All adult patients (age 18 and older) who had a healthcare encounter at Intermountain and a COPD diagnosis: ICD9 code (491.2, 492) in any sequence or a Diagnosis Related Group (DRG) (190–192) at any inpatient, Emergency Department, or ambulatory face-to-face encounter in 2013 or prior were identified (Supplementary Figure 1 and Supplementary Methods).

### Outcome variables

Mortality among derivation patients was assessed based on the known date of death in the Intermountain EHR for in-hospital deaths and was supplemented by Utah death certificate data and Social Security death master file records. Exacerbations requiring hospitalization and comorbidity rates were collected from the EHR.

### Clinical predictor variables

A complete list of variables is listed in Supplementary Table 1. We included a large number of variables in the dataset including PFTs. Due to the frequent cardiovascular comorbidities in COPD patients, and the likely contribution of fluid status in respiratory symptoms, we included a number of variables from Transthoracic Echocardiograms (TTE). We attempted to add 6-min walk distance and dyspnea scores, but these were not available in an encoded format in our data system.

### Statistical analysis

#### Cluster analysis

Hierarchical cluster analysis of the clinical variables was carried out in the R statistical program. Cluster analysis (23, 32) was run with the “cluster” package, using the “daisy” function, which calculates the Gower's distance for mixed variables (i.e., continuous and nominal variables). All of the vast arrays of clinical variables were included in the cluster analysis to determine the optimal clusters for a derivation subset of Intermountain patients with available data for most of the variables. Some variables were only available only for a minority of patients but cluster analysis is robust to missing data and can proceed with these variables included. We visually analyzed the cluster tree (dendrogram) and evaluated 4, 5, 6, 7, 8, and 9 cluster solutions using the “cutree” function in R (33). A seven-cluster solution was identified based on variable break points, cluster sizes, and the initial goal of identifying four to eight clusters (Supplementary Figure 2). This method provided segmentation of the population using clinically similar groupings that were derived independently of study outcome variables.

#### Recursive partitioning

After the cluster analysis, we used Recursive Partitioning (RP) (21, 34, 35) to identify a parsimonious subset of variables that best predict the cluster assignments and are more likely to be available for use in other populations. RP is a nonparametric regression approach for modeling relationships among variables, which allows for evaluation of a large number of mixed predictor variables (i.e., continuous, ordinal, and categorical) with missing values, as is often the case with EHR clinical measures. We categorized continuous laboratory variables based on laboratory determined clinical cutoffs (e.g., low, normal, high), because it created more stable decision trees, then ran RP in the R statistical platform using the “rpart” package.

We attempted alternative statistical methods, such as stepwise regression analysis techniques, however due to the frequency of missing data in the dataset too many cases were eliminated in the modeling process. For example, given the large number of variables in the data set, almost no cases had all data elements (pulmonary function test data, medication, labs, healthcare utilization, echocardiograms, etc.).

We evaluated the concordance between our clusters and the RP tree assigned groups for all patients with complete data that allowed RP assignment. Then, we evaluated the concordance between clusters and RP assigned groups for patients with missing data where we imputed normal values. The RP assigned groups were named the LIVE Scores for those patients.

### Validation

We validated the LIVE Score and our findings internally within the Intermountain Healthcare system in an expanded cohort of 48,871 patients. External validation was done at two independent sites: 83,134 patients in the United States Veterans Affairs (VA) nationwide healthcare system EHR data (VA Informatics and Computing Infrastructure, VINCI) (36) and 3,236 the University of Chicago Medicine system (Supplementary Table 2 and Supplementary Figures 4–6). To validate the COPD clusters, we used the RP tree derived above to empirically assign LIVE Scores based on the limited number of variables needed for the tree. This approach allowed us to validate the tree in external sites based on a much smaller number of variables. Kaplan–Meier survival curves were calculated to evaluate time to event results for mortality and exacerbation outcomes.

## Results

### Subject demographics

From the initial 11,048 patients identified with a COPD diagnosis in the Intermountain Healthcare system on or before 2013, the presence of a transthoracic echocardiogram (TTE), not its findings, was the initial most important variable for risk stratification. This observation suggested selection bias and pattern of care: patients who were more likely to come to the hospital often were more likely to get a TTE. Thus, we decided to focus on the higher risk patients (those with a prior TTE) for our cluster analysis.

### Cluster analysis

Cluster analysis of the 5,006 patients with a COPD diagnosis and a TTE by 2013 was performed using all clinical variables. A seven-cluster solution was identified based on variable break points, cluster sizes, and the initial goal of identifying four to eight clusters (Supplementary Figure 2). The seven clusters differed in number of patients, overall mortality, and healthcare utilization data (Supplementary Cluster Descriptions, Supplementary Tables 3, 4). We had encoded PFT data available for only 11% (535) of patients in our cohort, and the vast majority had obstruction (Supplementary Figure 6).

### Recursive partitioning and tree diagram

**Figure 1:**
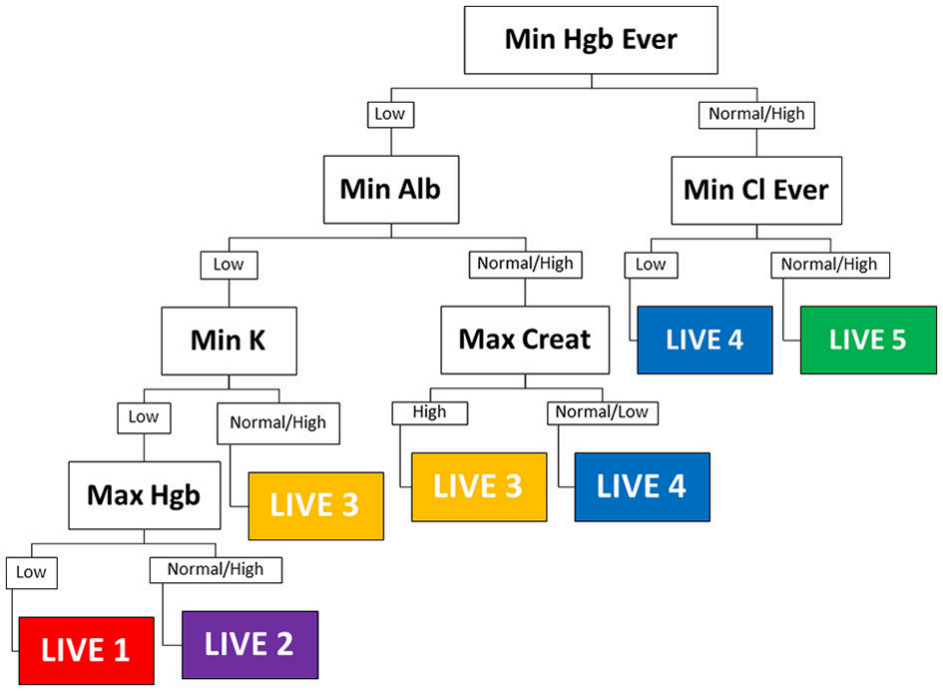
Decision tree. The empiric decision tree assigning five LIVE Scores of the seven cluster types is shown. Six laboratory variables categorize all patients into one of five LIVE Scores (approximately corresponding to the clusters). LIVE 5 (cluster 2), the “healthiest” is characterized by normal hemoglobin and normal chloride. LIVE 1 and 2 (clusters 1 and 6) the “sickest” are characterized by multiple laboratory abnormalities—most notably hemoglobin, albumin, and potassium. The presence of history of renal failure (Max Creat is high) distinguishes the higher risk LIVE 3 (cluster 5) from the relatively lower risk LIVE 4 (cluster 3). Max, maximum; Min, minimum; hgb, hemoglobin; creat, creatinine; Cl, Chloride, Alb, albumin; K, potassium; nl, normal; “ever” −4/1/2004 to 12/31 of current year. Final date for the datasets is 12/31/2014; when time not indicated—it means for the current year—in this case for 1/1/2013 to 12/31/2013.

We used Recursive Partitioning to derive an empiric decision tree assigning each patient into a specific LIVE Score (Figure 1). The decision tree had six nodes: albumin, creatinine, chloride, potassium, and hemoglobin (two variables: the minimum hemoglobin value over all years in the dataset, and the maximum hemoglobin value for the year). Using these six variables, the decision tree assigned each subject to one of five LIVE Scores. The decision tree did not assign two original cluster types (Cluster 4, *n* = 251, 5% and Cluster 7, *n* = 79, 1.6%). The agreement between the RP decision tree assigned LIVE Scores and the original Clusters is shown in Supplementary Figures 7–9.

### Validation

#### Intermountain validation

We identified all Intermountain Healthcare patients with a billing code for COPD based on an expanded list of COPD diagnosis codes to create a dataset of 48,871 patients alive in 2009 (Supplementary Table 2 and Supplementary Figure 3). Thirty thousand five hundred and thirty-three patients had laboratory data allowing LIVE Score calculation without imputing: basic demographics, healthcare utilization, and comorbidity rates for the 9,221 patients with a TTE in 2009 or prior (Tables 1, 2) and for the 21,312 patients without a TTE (Supplementary Tables 5, 6) are summarized.

**Table 1:** Demographics and clinical characteristics for patients with a prior transthoracic echocardiogram (TTE) and laboratory variables allowing LIVE Score assignment in 2009 (*N* = 9221).

**Variable name**	**Total**	**LIVE Score 5**	**LIVE Score 4**	**LIVE Score 3**	**LIVE Score 2**	**LIVE Score 1**	***P*-value**
Number of patients, *N* (%)	9,221	1,971 (18)	2,964 (28)	2,839 (26)	1,125 (11)	322 (3)	<0.001
Age, mean (*SD*)	67 (13)	64 (14)	66 (13)	71 (12)	67 (14)	70 (12)	<0.001
Female, *N* (%)	4,539 (49)	1,023 (52)	1,528 (52)	1,232 (43)	670 (60)	86 (27)	<0.001
White, *N* (%)	8,187 (89)	1,797 (91)	2,664 (90)	2,505 (88)	958 (85)	263 (82)	<0.001
8-yr mortality *N* (%)	4,283 (46)	457 (23)	1,185 (40)	1,696 (60)	697 (62)	248 (77)	<0.001
**Healthcare Utilization**							
ED & Inpatient COPD visits/year, mean (*SD*)	0.67 (1.2)	0.20 (0.6)	0.45 (0.9)	0.82 (1.3)	1.46 (1.6)	1.57 (2.0)	<0.001
ED COPD visits/year, mean (*SD*)	0.18 (0.6)	0.10 (0.4)	0.18 (0.6)	0.21 (0.7)	0.26 (0.7)	0.28 (0.9)	<0.001
Inpatient COPD visits/year, mean (*SD*)	0.49 (0.9)	0.10 (0.3)	0.27 (0.6)	0.62 (0.9)	1.20 (1.2)	1.30 (1.4)	<0.001
Outpatient COPD visits/year, mean (*SD*)	0.52 (1.3)	0.41 (1.1)	0.56 (1.3)	0.56 (1.5)	0.52 (1.6)	0.41 (1.1)	0.001
ED & Inpatient Any Cause Visits/year, mean (*SD*)	1.80 (2.5)	0.76 (1.4)	1.40 (2.3)	2.09 (2.4)	3.54 (3.0)	3.12 (3.1)	<0.001
ED any cause visits/year, mean (*SD*)	0.88 (1.9)	0.54 (1.3)	0.88 (2.0)	0.95 (1.9)	1.25 (2.1)	0.98 (2.0)	<0.001
Inpatient any cause visits/year, mean (*SD*)	0.92 (1.3)	0.22 (0.5)	0.52 (0.8)	1.14 (1.2)	2.3 (1.7)	2.14 (1.7)	<0.001
Outpatient any cause visits/year, mean (*SD*)	5.24 (6.2)	3.97 (4.5)	5.28 (5.7)	6.00 (7.1)	5.63 (7.3)	4.65 (6.7)	<0.001

*N, Number; % (percent); ED, Emergency Department; yr, year; SD, standard deviation; COPD, chronic obstructive pulmonary disease; &, and*.

**Table 2:** Comorbidities and laboratory results for patients with a prior transthoracic echocardiogram (TTE) and laboratory variables allowing LIVE Score assignment in 2009 (*N* = 9,221).

**Variable name, *N* (%)**	**Total**	**LIVE Score 5**	**LIVE Score 4**	**LIVE Score 3**	**LIVE Score 2**	**LIVE Score 1**	***P*-value**
Number of patients	9,221	1,971 (18)	2,964 (28)	2,839 (26)	1,125 (11)	322 (3)	<0.001
**CHARLSON COMORBIDITY RATE**							
Malignancy	1,876 (20)	222 (11)	579 (20)	689 (24)	277 (25)	109 (34)	<0.001
Diabetes	4,107 (45)	513 (26)	1,264 (43)	1,627 (57)	536 (48)	167 (52)	<0.001
Liver disease	1,983 (22)	284 (14)	618 (21)	675 (24)	317 (28)	89 (28)	<0.001
Chronic pulmonary disease	8,005 (87)	1,502 (76)	2,608 (88)	2,551 (90)	1,046 (93)	298 (93)	<0.001
Rheumatologic disease	1,027 (11)	132 (7)	358 (12)	353 (12)	152 (14)	32 (10)	<0.001
Myocardial infarction	2,724 (30)	323 (16)	779 (26)	1,127 (40)	369 (33)	126 (39)	<0.001
Cerebrovascular disease	2,971 (32)	494 (25)	932 (31)	1,050 (37)	368 (33)	127 (39)	<0.001
Chronic heart failure	5,254 (57)	612 (31)	1,637 (55)	2,041 (72)	741 (66)	223 (69)	<0.001
Dementia	358 (4)	30 (2)	88 (3)	148 (5)	75 (7)	17 (5)	<0.001
Peripheral vascular disease	3,195 (35)	397 (20)	1,012 (34)	1,238 (44)	422 (38)	126 (39)	<0.001
Renal disease	2,533 (28)	122 (6)	317 (11)	1,517 (53)	412 (37)	165 (51)	<0.001
Peptic ulcer disease	1,321 (14)	150 (8)	426 (14)	470 (17)	214 (19)	61 (19)	<0.001
**LABORATORY VALUE ABNORMALITIES**							
Max BNP ever high	4,846/6,303 (77)	415/823 (50)	1,405/2,002 (70)	1,956/2,287 (86)	823/920 (90)	247/271 (91)	<0.001
Max HbA1C ever high	3,198/5,030 (64)	387/691 (56)	989/1,594 (62)	1,294/1,867 (69)	407/688 (59)	121/190 (64)	<0.001
Max PCO2 ever high	3,555/4,938 (72)	200/433 (46)	1,093/1500 (73)	1,357/1831 (74)	694/914 (76)	211/260 (81)	<0.001
Max CO2 ever high	5,066/9,220 (55)	448/1,970 (23)	1,719/2,964 (58)	1,796/2,839 (63)	861/1,125 (77)	242/322 (75)	<0.001
Max eosinophil count ever high	2,297/9,085 (25)	216/1,891 (11)	661/2,938 (23)	910/2,819 (32)	386/1,118 (35)	124/319 (39)	<0.001
Max albumin ever high	747/9,132 (8)	130/1,892 (7)	282/2,954 (10)	230/2,839 (8)	93/1,125 (8)	12/322 (4)	<0.001
Max creatinine ever high	5,268/9221 (57)	511/1,971 (26)	1,148/2,964 (39)	2,539/2,839 (89)	828/1,125 (74)	242/322 (75)	<0.001
Min Hgb ever low	6,592/9,221 (72)	0/1,971 (0)	2,306/2,964 (78)	2,839/2,839 (100)	1,125/1,125 (100)	322/322 (100)	<0.001

*N, Number; % (percent); ED, Emergency Department; yr, year; SD, standard deviation; max, maxiumum; min, minimum; BNP, B-type Natriuretic Peptide; HbA1c, hemoglobin A1C or glycohemoglobin; PCO2, partial pressure of carbon dioxide in the arterial blood; Hgb, hemoglobin*.

**Figure 2:**
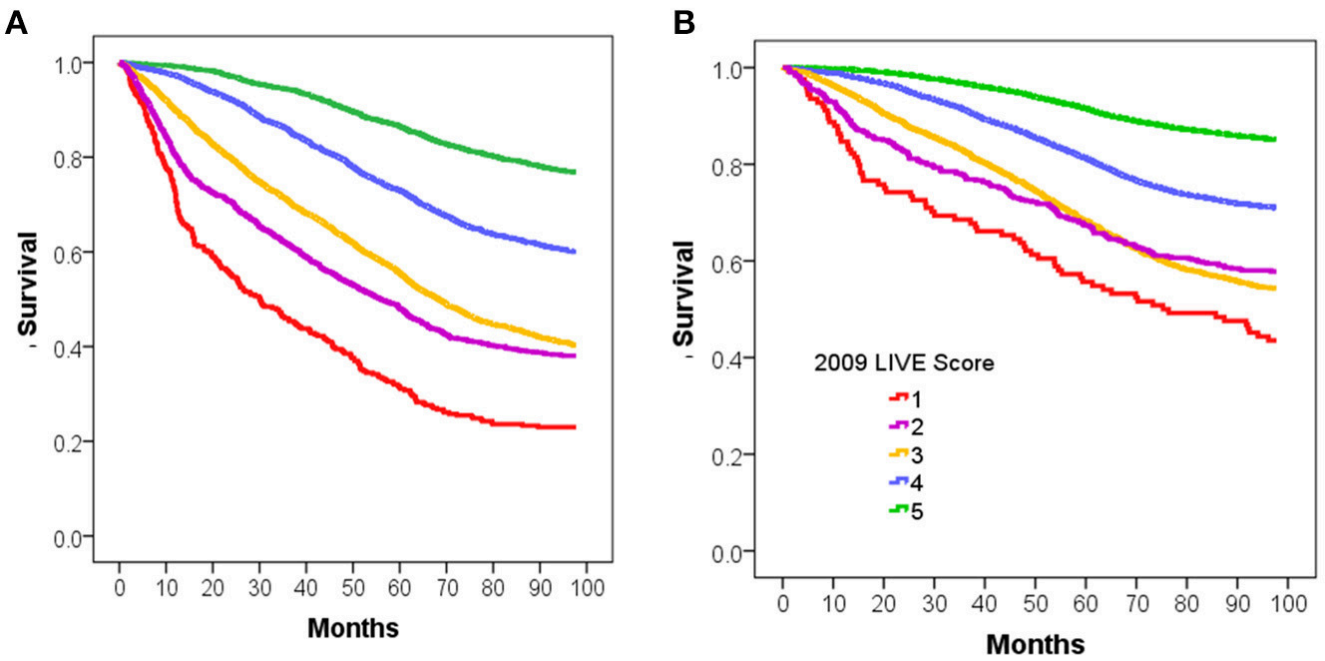
Kaplan-Meier survival analysis for intermountain validation cohort. Figure shows the Kaplan-Meier survival analysis for 8 year mortality by LIVE Score assignment in 2009 for patients at Intermountain. **(A)** 8-year mortality for the 9,221 patients with Transthoracic echocardiogram (TTE) in 2009 with no missing data is shown. The LIVE Score separates patients by mortality with the lowest mortality in LIVE Score 5, increasing with LIVE Score 4, 3, and 2, and LIVE Score 1with the highest mortality (*p* < 0.001). **(B)** The same pattern of increasing mortality with decreasing LIVE Score in the 38135 patients without a TTE in 2009 or prior is shown.

Overall mortality was assessed for each of those cohorts based on the calculated LIVE Score in 2009. The mortality for patients with a TTE was higher than the mortality for patients without a TTE (46 vs. 23%, respectively, *p* < 0.001), and the LIVE Scores stratified mortality within both cohorts. Figure 2 shows the Kaplan–Meier survival curve for patients with (Figure 2A) and without a prior TTE (Figure 2B). In both cohorts, LIVE Score 5 had the lowest mortality (23 and 15%, respectively, *p* < 0.001) and LIVE Score 1 (77–57%, *p* < 0.001) had the highest mortality (Figure 2).

**Figure 3:**
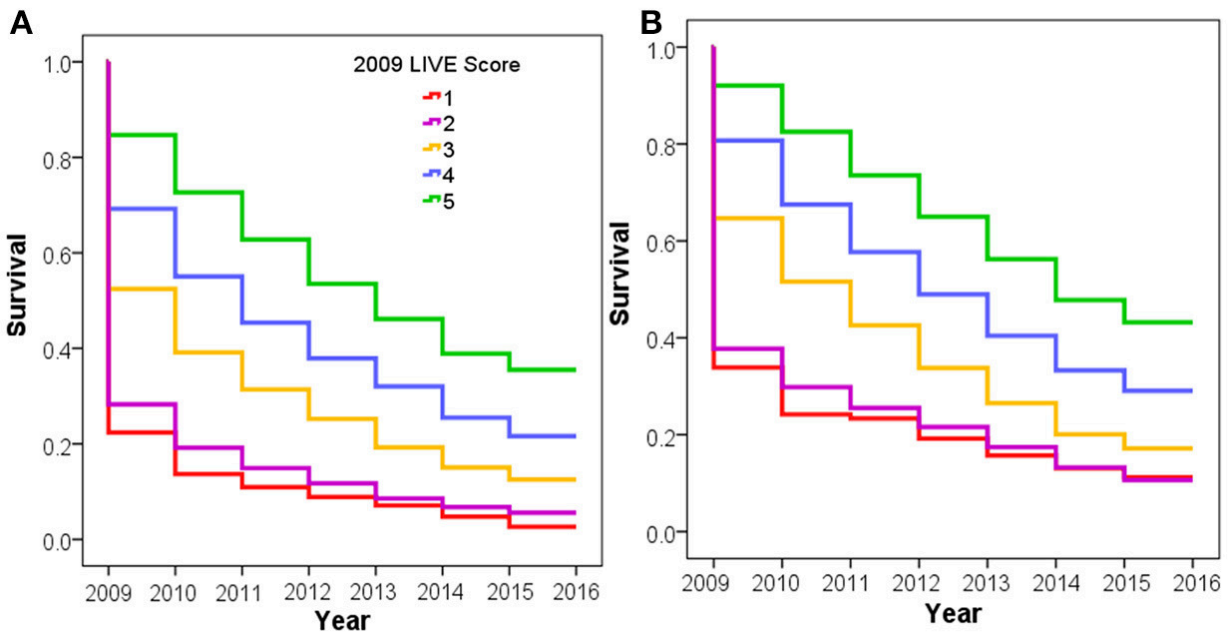
COPD exacerbation rate by LIVE Score. Figure shows increasing COPD exacerbation rates with decreasing LIVE Score in the Intermountain cohort. **(A)** Increasing COPD exacerbation risk for the 9,221 patients with a TTE in 2009 or prior lab data in 2009 allowing LIVE Score assignment is shown. **(B)** The same pattern of increasing risk of COPD exacerbation rates for the 38,135 patients without a prior TTE is shown.

The time to first COPD exacerbation requiring a COPD-related Emergency Department visit and/or hospitalization was also statistically significantly different in both cohorts (Figure 3). Patients with LIVE Score 5 with a prior TTE had the lowest rate of COPD exacerbations (0.20 COPD related visits/year vs. 0.67 visits/year overall, *p* < 0.001). Patients with LIVE Scores 1 and 2 had the highest COPD related healthcare utilization rate (1.57 and 1.46 visits/year, respectively). The difference between COPD exacerbations with LIVE Score 1 and LIVE Score 2 was not significant, but both were statistically significantly higher compared with the overall rate of 0.20 visits/year, *p* < 0.001 (Table 1 and Figure 3).

The LIVE Scores with higher overall mortality were statistically significantly associated with higher comorbidity rates (Tables 1, 2, and Supplementary Tables 5, 6).

#### Veterans affairs national health system validation

**Figure 4:**
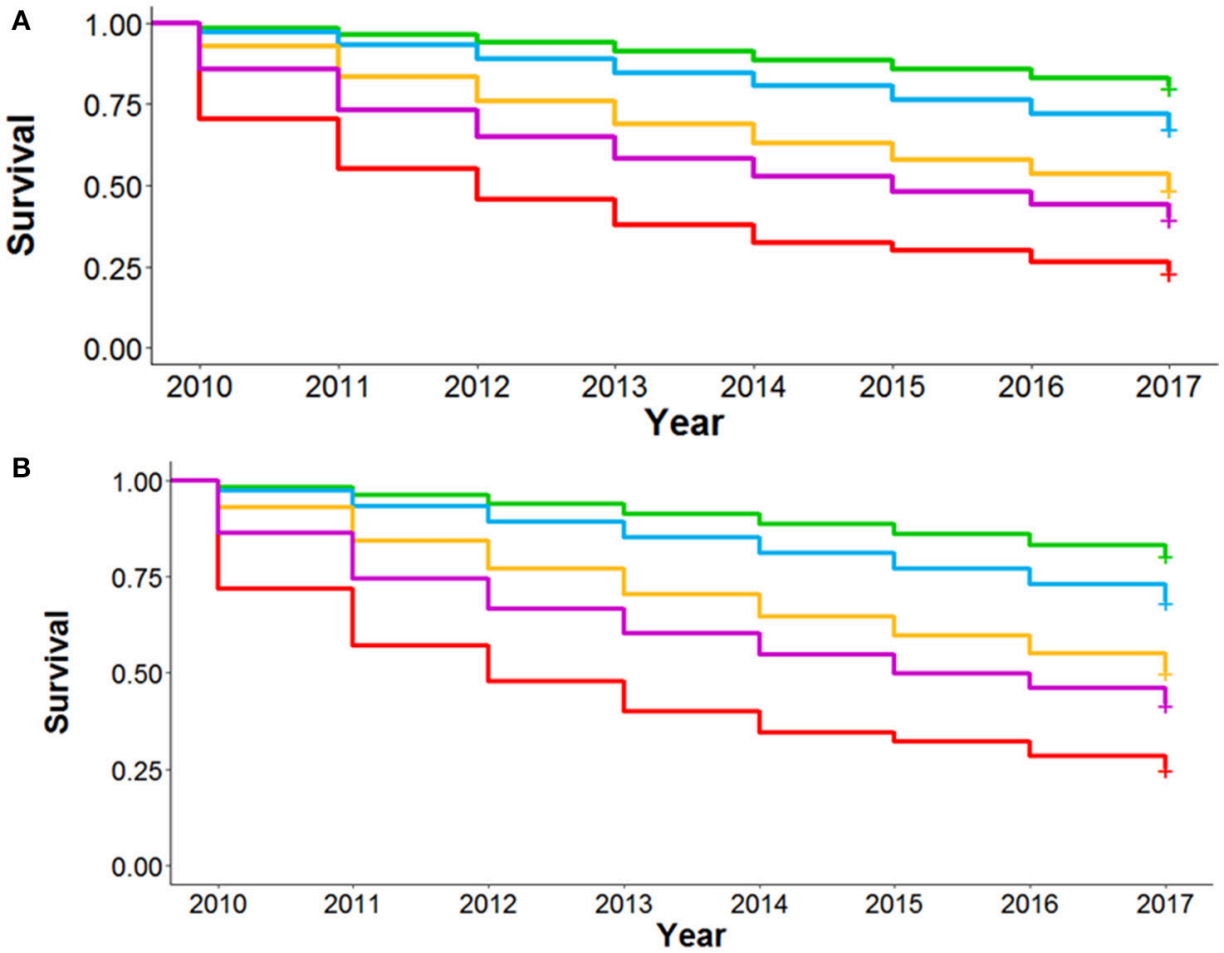
Kaplan-Meier survival analysis for National Veterans Affairs Health System Validation Cohort. Figure shows the Kaplan-Meier survival analysis for 8 year mortality by LIVE Score assignment in 2009 for patients at the National Veterans Affairs Health System. **(A)** 8-year mortality for the 6,034 patients with Transthoracic echocardiogram (TTE) in 2009 with lab data allowing RP assignment of LIVE Score in 2009 is shown. The LIVE Score separates patients by mortality with the lowest mortality in LIVE Score 5, increasing with LIVE Score, 3, 2, and LIVE Score 1 with the highest mortality (*p* < 0.001). **(B)** The same pattern of increasing mortality with decreasing LIVE Score in the 77,100 patients without a TTE in 2009 or prior is shown.

External Validation was performed in a retrospective data-only cohort of 83,134 VA patients with COPD alive in 2009 from all VA hospitals throughout the United States who had a LIVE Score calculation in 2009 (Supplemental Table 7 and Supplementary Figure 4). We performed the analysis on the 6,034 patients who had TTE in 2009 or prior and examined 7-year mortality and risk of severe COPD exacerbation. We repeated the analysis on the 77,100 patients without a TTE in 2009 or prior. Patients with a prior TTE had a statistically significantly higher overall mortality than those without a prior TTE (Supplementary Figure 12). However, within each cohort the LIVE Score separated patients into statistically significantly different overall mortality rates (Figure 4). LIVE Score 1 patients with a prior TTE had an 81% mortality compared with 23% mortality for LIVE Score 5 patients (*p* < 0.001 Figure 4A). Similarly, LIVE Score 1 patients without a prior TTE had a 72% mortality compared with 17% mortality for LIVE Score 5 (*p* < 0.001, Figure 4B). Furthermore, in both cohorts, the LIVE Scores were associated with statistically significantly different rates of COPD exacerbation. The highest rates were in patients with LIVE Scores 1 and 2 where 80–84% of patients had a COPD exacerbation by 8 years, respectively. Although the difference between LIVE Score 1 and 2 was not significant, both groups were statistically significantly higher compared with the other LIVE Scores and only 25% of patients in LIVE Score 5 had a COPD exacerbation (*p* < 0.001, Supplementary Figures 13, 14).

#### University of chicago health system validation

We repeated the LIVE Score validation in a second retrospective data-only cohort of 3,236 patients from the University of Chicago Medicine system where TTE data were not available (Supplementary Tables 2, 8 and Supplementary Figure 5). The University of Chicago Medicine system is relatively open and comprises a unique urban population. Patient cohort enrollment was normalized such that time zero for patient data was the date that patients first met cohort criteria. Given the relatively small number of patients in the cohort, as well as the Intermountain work showing good predictions when imputing missing variables as normal (Supplementary Figures 9, 10), we elected to impute missing variables as “normal” in this cohort.

**Figure 5:**
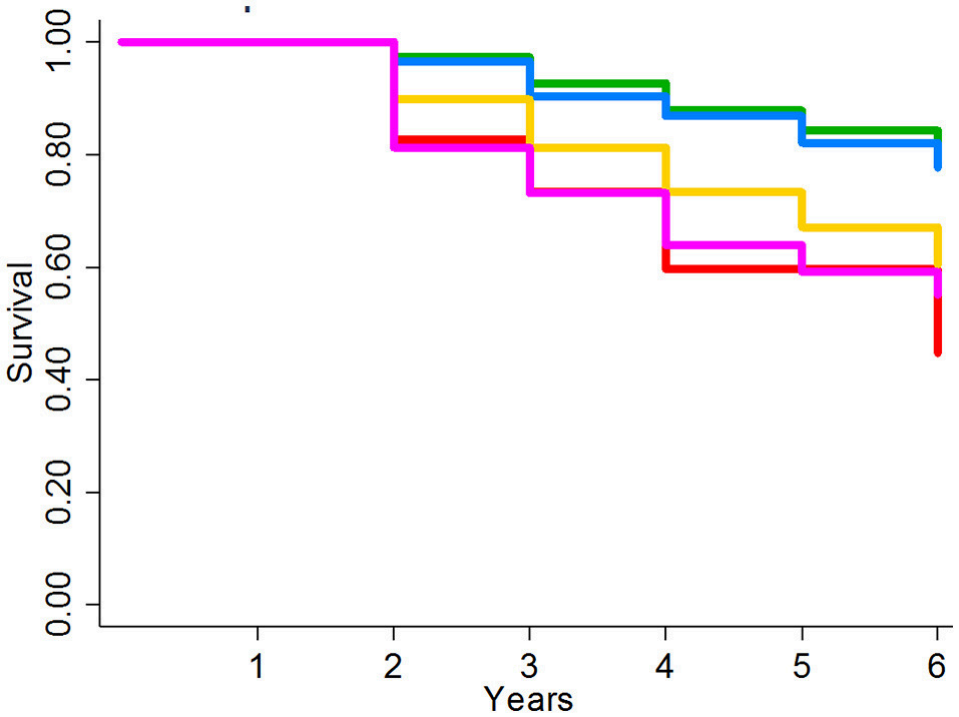
Kaplan-Meier survival analysis for University of Chicago Health System Validation Cohort. Figure shows the Kaplan-Meier graph for 6 year overall mortality for 3236 patients at the University of Chicago patients with unknown TTE data. The lowest risk groups (LIVE Scores 4 and 5) and the highest risk groups (LIVE Scores 1, 2, and 3) show significant spread in mortality. In this small cohort in an open system without known TTE data, no significant difference was found within the low-risk LIVE Scores (4 and 5) and the high-risk LIVE Scores (3 vs. 2 vs. 1).

In this second external cohort with slightly different COPD definitions and a unique patient population, the LIVE Score showed the same pattern of separation of 6-year all-cause mortality (Figure 5). Overall the separation was between two low-risk LIVE Scores (LIVE Score 4 and 5) and three high-risk LIVE Scores (LIVE Scores 1, 2, and 3). The difference between the low risk and high-risk LIVE Scores was statistically significant, but in this small cohort of patients the differences among the individual LIVE Scores did not reach statistical significance (Supplementary Table 9). Similarly, in this small cohort with a large number of imputed variables in a relatively open health system, differences among the LIVE Scores with regard to severe COPD exacerbation were not found to be statistically significant (Supplementary Table 10 and Supplementary Figure 15).

## Discussion

Using a large dataset of routinely collected clinical variables from the EHR and narrowing it down to an optimal parsimonious set of common variables, we identified and externally validated a novel Laboratory-based Intermountain Validated Exacerbation (LIVE) Score in patients diagnosed with COPD. The LIVE Score is calculated based on six routinely collected laboratory values, which are reliable across institutions and care settings, are obtained in real time, and do not rely on clinician judgment or billing codes. The LIVE Scores stratify patients with differing overall mortality rates and severe COPD exacerbation rates across different healthcare systems.

The LIVE Score is based on the hemoglobin, potassium, albumin, creatinine, and chloride laboratory values obtained through routine clinical care. Although our analysis does inform why these specific variables most robustly separated patients with a diagnosis of COPD into different groups, we speculate that they may be markers of comorbidity and disease. For example, patients who not had evidence of renal failure (maximum creatinine is normal) would be at lower risk for complications related to congestive heart failure exacerbations and would be less likely to be hospitalized or to die. Additionally, those with evidence of anemia (minimum hemoglobin ever low), may be a marker for patients with anemia of chronic disease, which in turn may be related to their other morbidies and the patient's overall health. Similar speculations regarding the correlation of mortality and laboratory abnormalities may be made regarding potassium (e.g., diuretic use), albumin (malnutrition, general health), or chloride.

The value of risk stratifying patients based on the LIVE Score lies in the ability to identify high-risk patients across a healthcare system for targeted interventions. Although the bedside physician may recognize that their individual COPD patient is at high risk for mortality and future healthcare utilization, identifying high-risk patients on a system level allows for resource allocation that would better support the patient and their physicians. Indeed, while large gaps between recommended care and actual care in COPD patients remain (37–40), this type of risk stratification may help improve adherence to guidelines in the high-risk patients who need better support. Thus, the utility of risk stratification is that within a health system identifying high-risk patients may help focus resources around improving access to care and care coordination (41–43). This approach of risk stratifying patients based on passively collected and calculated risk scores with subsequent intensive clinician attention to the highest risk patients has been shown to be effective in improving heart failure and sepsis outcomes (30, 31).

The LIVE Score risk stratifies complex real-world patients who have been diagnosed with COPD and may have a variety of competing comorbidities, which affect their overall mortality and healthcare utilization. These comorbidities are important determinants not only of overall mortality, but also of hospitalizations and healthcare utilization. While healthcare systems have increased their focus on reducing 30-day COPD readmissions, nearly half of the patients readmitted after a COPD related hospitalization are admitted for problems unrelated to their COPD (43). Thus, interventions aimed at improving COPD care must take into account the multimorbidity model of COPD in identifying patients (19, 43, 44). Indeed, for many patients with COPD, improving care may be achieved more effectively by diagnosing and treating comorbidities rather than focusing on COPD therapy alone (45).

The strength of our study is the empiric, reliable, risk stratification of COPD patients using readily available EHR data. The validation using clinical patient data from three different healthcare systems with different definitions of COPD suggests that these groups reflect underlying stable patient groups. This risk stratification strategy may form a basis for identifying COPD patients at high risk of mortality and complications on a system level thus better targeting interventions. Although our study advances the field by identifying novel laboratory based LIVE Scores in COPD patients, it has some limitations. First and foremost, unlike research cohorts with prospectively collected PFT data, we cannot be certain that all patients have COPD. This limitation in identifying and categorizing COPD patients reflects the underlying structure of most EHR systems, which do not have PFTs available, and system limitations whereby patients with COPD do not regularly receive PFT testing. Nevertheless, factors beyond PFTs are increasingly recognized as driving outcomes in patients with COPD (4). The lack of diagnostic certainty does not take away from the utility of our LIVE Scores. Our cohorts represent patients in clinical care with diagnostic uncertainty and competing comorbidities, which may cause respiratory symptoms that are evaluated in routine clinical care. Indeed, our risk stratification schema may facilitate more accurate diagnosis of COPD by prioritizing diagnostic accuracy in high-risk patients where additional resources may be focused.

## Conclusion

In large clinical datasets across different organizations, a LIVE Score that utilizes existing laboratory data for COPD patients may be used to stratify risk for mortality and COPD exacerbations.

## IMPACT

Despite advancements in interventions that improve clinical outcomes of COPD patients, gaps between clinical guidelines and care persist. While COPD patients in clinical research studies are well-characterized and managed according to current guidelines, in clinical care those hospitalized with respiratory symptoms may have diagnostic uncertainty and lack guideline recommended care. Identifying the highest-risk groups of COPD patients in order to prioritize enrollment in disease management programs remains a challenge. Here we developed and validated the LIVE Score, a system for population health management to identify COPD patients at high risk for healthcare utilization, morbidity, and mortality through existing data for real-world clinically diagnosed COPD. The LIVE Score could be used to risk stratify COPD patients within a healthcare system in order to prioritize initiatives aimed at improving healthcare delivery for COPD, saving clinician time and reducing health system costs.

## Author contributions

DB conceived of the study, designed the data set, performed data analysis and interpreted the data, and wrote the first draft of the manuscript. DB had full access to the data and is the guarantor of the paper, taking responsibility for the integrity of the work as a whole, from inception to published article. DC helped design the study, performed the statistical analysis and validation, and critically revised the manuscript for important intellectual content. SR generated and validated the dataset, helped analyze the data, and critically revised the manuscript for important intellectual content. BH helped analyze the data and critically revised the manuscript for important intellectual content. VP, MC, and KC validated the findings in the University of Chicago data and edited the manuscript. RM assisted with data analysis and interpretation, edited the manuscript. SZ generated the data set and R code and validated the dataset in the VA data set and edited the manuscript. MA generated the data and analyzed the data for the validation in the VA cohort and critically revised the manuscript for important intellectual content.

### Conflict of interest statement

BH is supported by grants from CareCentra, GlaxoSmithKline, and AstraZeneca for the development and/or clinical implementation of clinical decision tools. The remaining authors declare that the research was conducted in the absence of any commercial or financial relationships that could be construed as a potential conflict of interest. The reviewer NK and handling Editor declared their shared affiliation.
