# Supplementary material for: The Laboratory-Based Intermountain Validated Exacerbation (LIVE) Score Identifies Chronic Obstructive Pulmonary Disease Patients at High Mortality Risk
Source: Front Med (Lausanne). 2018 Jun 11;5:173. doi: 10.3389/fmed.2018.00173 (PMC6004514; doi:10.3389/fmed.2018.00173)
Supplement: Supplementary file 1 [file Data_Sheet_1.docx]

20171204 Supplementary Figures and Tables Frontiers in Medicine: Pulmonary Medicine

**The Laboratory-based Intermountain Validated Exacerbation Score Identifying High Risk Chronic Obstructive Pulmonary Disease Patients**

**Running Title:** LIVE Score COPD Risk Stratification

**Please note: Figures in manuscript are re-presented here with more detailed statistics. They are presented without additional statistics in manuscript for ease of reading.**

**Figure 2 from Main Manuscript:**

**
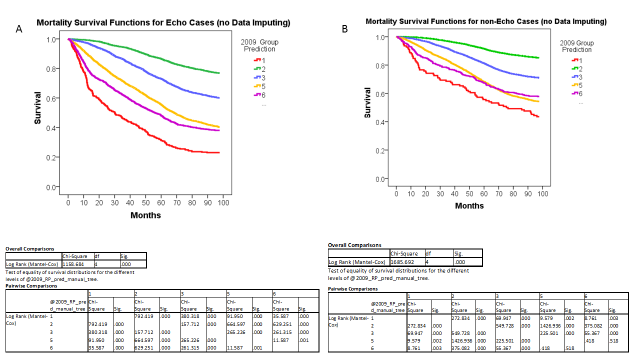
**

**Figure 3 from Main Manuscript:**

**
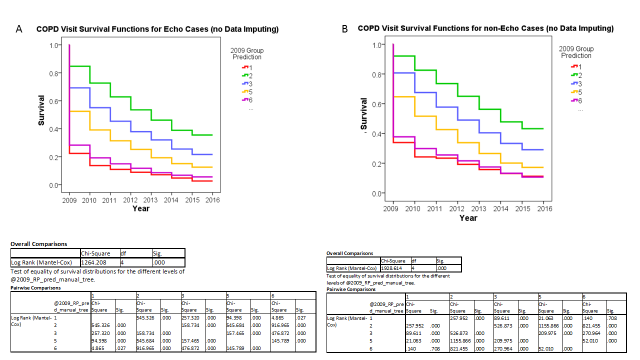
**

**Figure 4 from Main Manuscript:**

**
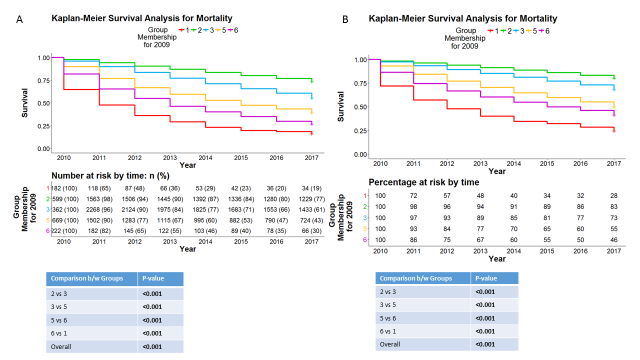
**

**Supplementary Methods**

**Outcome Variables**

Mortality among derivation patients was assessed based on the known date of death in the Intermountain EHR for in-hospital deaths and was supplemented by Utah death certificate data and Social Security death master file records. Exacerbations requiring hospitalization and comorbidity rates were collected from the EHR. Prior experience indicates that >90% of patients return to the 24 Intermountain hospitals and 185 clinics for subsequent care (unpublished data), thus loss to follow up for non-fatal events is reliable.

**Clinical Predictor Variables**

A complete list of variables is listed in Supplementary Table 1. We included a large number of variables in the dataset that we hypothesized would be important in COPD. Due to the frequent cardiovascular comorbidities in COPD patients, and the likely contribution of fluid status in respiratory symptoms, we included a number of variables from Transthoracic Echocardiograms (TTE’s), including the ejection fraction (EF), measures of pulmonary hypertension (cor pulmonale) such as the Right Ventricular Systolic Pressure (RVSP) and Tricuspid Annular Plane Systolic Excursion (TAPSE) from an existing TTE database in our system.

Charlson comorbidity rates are routinely collected as part of the EHR and were included. A large number of other variables were included – not only the complete blood count with differential (CBC with Diff) (including eosinophils) and complete metabolic panel (CMP), but also hemoglobin A1c, Brain-type natriuretic peptide (BNP), and arterial blood gas (ABG) values. We included the partial pressure of carbon dioxide (pCO2) and also a derived delta pCO2 (difference of the minimum and maximum pCO2 within the year, postulating possible association with exacerbations or intubation). Similarly, we included carboxyhemoglobin levels as a possible surrogate for active tobacco use. For laboratory values where patients had multiple values within the same year, we used variables for the minimum value within the year, the maximum value within the year, the minimum value within the EHR, and the maximum value within the EHR.

We included all known PFT variables from our PFT database, which included the Forced Expiratory Volume in 1 second (FEV1) and FEV1 % predicted, the Forced Vital Capacity (FVC), the FEV1/FVC ratio as well as FVC, Forced Expiratory Volume in 6 seconds (FEV6), Diffusion capacity of the Lung for Carbon Monoxide (DLCO), Total Lung Capacity (TLC), and post-bronchodilator values. We also determined whether spirometry had been done outside our PFT database based on billing codes and included data on whether spirometry had been done within the previous year or ever. Outpatient respiratory inhaler use was determined based on pharmacy records and inpatient hospital records. We attempted to add 6-minute walk distance and dyspnea scores, but these were not available in an encoded format in our data system.

**Internal Validation**

We validated our findings in a large cohort of 48,871 patients in Intermountain Healthcare. We identified all Intermountain Healthcare patients with an expanded set of ICD-9 and ICD-10 billing codes for COPD (Supplementary Table 2) between 2009 and 2016. Intermountain is an integrated health system comprising 24 hospitals and 185 clinics in Utah and the surrounding states with a relatively stable patient population and relatively low rates of migration. Intermountain Healthcare serves a large rural population in Utah.

The dataset included the CBC with diff, CMP, the presence or absence of a prior TTE, mortality, healthcare utilization (COPD specific and overall), and Charlson Comorbidity rates. We used the preferred decision tree to assign patients to a LIVE Score based on the limited set of variables from the tree and evaluated eight year any cause mortality and COPD-related healthcare utilization.

**External Validation – National Veterans Affairs Health System**

We validated the LIVE Scores in the National Veterans Affairs (VA) Health System in a large cohort of 83,134 patients. All patients with COPD from all VA hospitals throughout the United States between 2009 and 2016 were included in the cohort with all tree variables to allow LIVE Score assignment. The cohort included data on overall mortality and COPD related healthcare utilization. The National Veterans Affairs Health System is a large national health system that is relatively closed. Many veterans who receive care at VA hospitals continue to present for subsequent follow up there preferentially.

**External Validation – University of Chicago Medicine System**

We validated the LIVE Scores in a small cohort of 3,236 COPD patients in the University of Chicago Medicine System. The University of Chicago Medicine System serves a unique urban patient population and is relatively open with a large number of patients who seek care at other health systems in the city. A cohort that included patients with a history of COPD based on billing codes after their first COPD exacerbation hospitalization were included. Missing variables were imputed as normal to allow LIVE Score assignment through the decision tree. Missing variables were imputed as “normal” when patients had no values for the entire year and values were carried forward for missing “ever” variables. No imputation of variables was done for years after the last year of real data. Severe COPD exacerbations and overall mortality data were tracked based on time since entering the cohort, rather than by specific calendar year.

**Supplementary Results
Description of Clusters**

**Cluster 5**

Cluster 5 comprised 16% of subjects (774 of 5006) with the lowest overall four year mortality (8% vs. 29% overall, p<0.001). Patients in Cluster 5 were younger (mean age 64 vs. 70 overall, p<0.001), had the lowest healthcare utilization (inpatient, ED, and overall for COPD-related visits and any cause) and had the lowest rate of comorbidities. The mean rate of annual severe COPD exacerbations (requiring ED visit and/or hospitalization) was 0.12 visits/year vs. 0.34 visit/year for the group overall. They had the lowest rate of diabetes (20% vs. 45% overall, p<0.001), congestive heart failure (CHF) (25% vs. 58% overall, p<0.001), and other comorbidities (Supplementary Table 4). Thus Cluster 5 selected younger, healthier patients with the lowest mortality and the lowest healthcare utilization rates.

**Cluster 4**

Cluster 4 selected 1217 patients (24% of all) with the second lowest four year mortality (17% vs. 29% overall, p<0.001), below average COPD related healthcare utilization rates (0.23 visits/year vs. 0.34 visits/year overall, p<0.001), and relatively low rates of comorbidity (e.g. 42% prevalence of diabetes vs. 45% overall, p<0.001, 47% prevalence of CHF vs. 58% overall, p<0.001).

**Cluster 3**

The 1499 patients that comprised Cluster 3 (30% of the cohort) formed the largest cluster. These patients were the oldest (mean age 74.5 years old vs. 70 years old overall, p<0.001) and had a four year mortality higher than the overall group (35% vs. 29%, p<0.001) with below average healthcare utilization rates (0.24 visits/year vs. 0.34 visits/year overall, p<0.001). Cluster 3 patients had a high rate of comorbidities including 53% prevalence of diabetes (vs. 45% overall, p<0.001), 73% prevalence of CHF (vs. 58% overall, p<0.001) among others (Supplementary Tables 3 and 4).

**Clusters 1 and 2**

Clusters 1 and 2, among the smallest sized clusters that comprise 13% (662 patients) and 11% (524 patients) of the population, respectively, select the highest risk patients. Cluster 2 and Cluster 1 have the highest four year mortality rates (54% and 62%, respectively vs. 45% overall), the highest healthcare utilization rates (COPD-specific and overall) (0.76 and 0.69 COPD related visits/year, respectively, vs. 0.34 visits/year overall, p<0.001), and the highest comorbidity rates (65% and 83% CHF prevalence vs. 58% overall, p<0.001, and 54% and 62% diabetes prevalence vs. 45% overall, p<0.001). (See Supplementary Tables 3 and 4).

**Clusters 6 and 7**

Clusters 6 and 7 were small (comprising only 5% - 251 patients and 2% - 79 patients, respectively) with below average four year mortality (24% for both vs. 29% overall, p<0.001), and average healthcare utilization and comorbidity rates (Supplementary Tables 3 and 4).

**Decision Tree Assigned LIVE Score Concordance with Cluster**

Of the 5,006 patients in 2013, all variables were available to assign a LIVE Score via the RP tree for the majority (3898, 78%). The majority of patients were assigned correctly by the RP tree when all variables were available for a LIVE Score assignment (Supplementary Figures 7-9). For the 1108 patients with missing data, missing variables were imputed as normal and the patients were assigned a LIVE Score with the RP decision tree. The agreement between the RP decision tree LIVE Score assignment and the original cluster type for these patients is shown in Supplementary Figure 9.

**Supplementary Results Validation**

We validated our LIVE Score assignments in three distinct cohorts. The first cohort was an expanded Intermountain Cohort of 48,871 patients with a COPD diagnosis based on an expanded number of billing codes between 2009 and 2016 (Supplementary Table 2). Next, we validated our LIVE Score assignments in the National Veterans Affairs Health System in 83,134 patients. The Intermountain cohort and the VA Health System cohort are relatively closed systems with a large number of patients at various hospitals and clinics. Data on the presence of a TTE was available for those cohorts and patients were analyzed in subsets based on the presence or absence of a TTE at the time of entering the cohort in 2009. Overall mortality and severe COPD exacerbation were assessed for several years for all patients. Finally, we validated our findings in a small, urban cohort at the University of Chicago Health System of 3,326 patients. The University of Chicago system is more open and there was a high rate of missing variables, thus for that cohort missing tree variables were imputed as normal.

**Internal Validation**

Despite variations in how we defined COPD (billing codes and CONSORT diagrams summarized in Supplementary Table 2 and Supplementary Figures 3-5) and distinct populations in these three unique health systems, our findings were reproducible across systems. The LIVE Score assignment using the RP tree separated patients with a diagnosis of COPD into groups of variable overall mortality. Furthermore, in the large datasets at relatively closed health systems at Intermountain Healthcare and the Veterans Affairs Health System, the LIVE Scores were associated with differing rates of severe COPD exacerbations.

Overall mortality was assessed for each of those cohorts (with and without TTE) separately based on LIVE Score assignment in 2009. Although the eight year mortality for patients with a TTE was higher than the seven year mortality for patients without a TTE (46% vs. 23%, respectively, p<0.001), the LIVE Scores stratified both cohorts based on overall mortality. Figure 2 shows the Kaplan-Meier eight year survival curve for patients without missing lab variables with a prior TTE (Figure 2A) and without a prior TTE (Figure 2B). In both cohorts, those with and those without a prior TTE in 2009, LIVE Score 5 had the lowest mortality (23% and 15%, respectively, p<0.001). LIVE Score 4 had the second lowest mortality in both TTE and no prior TTE cohorts (40% and 29%, respectively, p<0.001), followed by LIVE Score 3 (60% and 46%, p<0.001), then LIVE Score 2 (62% and 42%, p<0.001), and LIVE Score 1 (77% and 57%, p<0.001), which had the highest mortality (Figure 2). We performed the same analysis using imputed normal variables for those patients with missing variables, and again, the RP tree stratified patients with differing mortality based on their 2009 LIVE Score assignment in the same pattern of risk with LIVE Score 5 having the lowest mortality (Supplementary Figures 10 and 11).

**Supplementary Figure 1. Consort Diagram of the Derivation Cohort at Intermountain Healthcare**


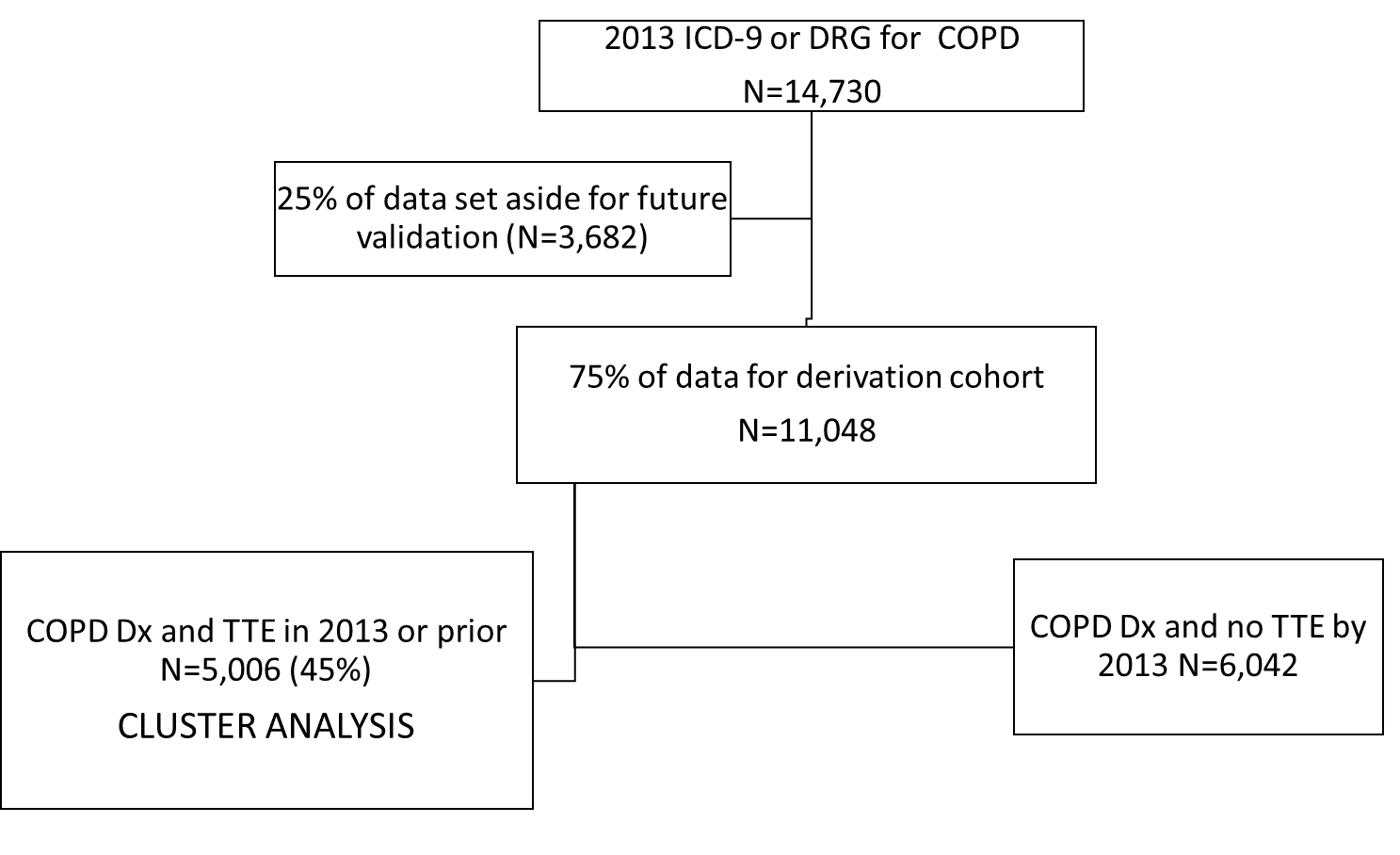


A consort diagram of the COPD cohort analyzed is presented. 25% of the initial dataset was set aside for internal validation for other findings. Of the remaining 11,048 patients, 45% (5,006) had a Transthoracic Echocardiogram (TTE) in 2013 or prior. Those 5,006 patients and their associated data were used for cluster analysis.

**Supplementary Table 1. Complete List of Variables in the Intermountain Healthcare Derivation Cohort.**

All variables are within the calendar year unless otherwise stated. All "ever" variables used 1980 as start year, however availability of values is as follows: start date 4/1/2004 for laboratory values, 1/1/2000 for Pulmonary Function Tests (PFTs), 1/1/2006 for ejection fraction (EF).

| **Supplementary Table 1. Complete List of Variables** | |
| --- | --- |
| **MORTALITY** | Mortality data were used in the cluster analysis but removed for recursive partitioning analysis |
| DTH_DT | death date |
| DTH_YR | death year |
| AGE_DTH | age at time of death |
| **MEDICATION CLASS PRESCRIBED**  BINARY - YES/NO; queried separately for each year between 2009 and 2014 | |
| MEDS_ICS | medications - inhaled corticosteroids |
| MEDS_XNTHNS | medications - xanthines |
| MEDS_PDE4 | medications - Phosphodiesterase 4 inhibitors |
| MEDS_LAMA | medications - long acting muscarinic antagonists |
| MEDS_SAMA | medications - short acting muscarinic antagonists |
| MEDS_LABA | medications - long acting beta agonists |
| MEDS_SABA | medications - short acting beta agonists |
| MEDS_BA_MA | medications LABA-LAMA combination |
| MEDS_BA_ICS | medications LABA-ICS combination |
| **DEMOGRAPHICS** | |
| smoking.status | smoking status by study year |
| Sex | sex |
| race | race |
| marital.status | marital status |
| avg.hshld.income | average household income by census zip code; non-Utah patients removed; |
| elevation | elevation by home address |
| Age | age by study year |
| **EJECTION FRACTION** | |
| min_EF_year_binary | Minimum Ejection Fraction within the calendar year; binary <40% or >=40% |
| max_EF_year_binary | Maximum Ejection Fraction within the calendar year; binary <40% or >=40% |
| min_EF_ever_binary | Minimum Ejection Fraction within the Electronic Health Record within the previous 5 years; binary <40% or >=40% |
| max_EF_ever_binary | Maximum Ejection Fraction within the Electronic Health Record within the previous 5 years; binary <40% or >=40% |
| **HEALTHCARE UTILIZATION** | |
| ED_COPD_VIS | Emergency Department visits for COPD within the calendar year (ICD9 491.2, 494 any sequence; DRG 190-192); counted only if not admitted, otherwise counted only as inpatient admission) |
| IP_COPD_VIS | Inpatient Admissions for COPD within the calendar year (ICD9 491.2, 492 any sequence; DRG 190 - 192) |
| OP_FF_COPD_VIS | Outpatient visits for COPD within the calendar year ( ICD9 491.2*, 492* any sequence) |
| OP_FF_ANY_VIS | Outpatient visits for any casue within the calendar year (must have at least one ICD code) |
| IP_ANY_VIS | Inpatient Admissions for any cause within the calendar year |
| ED_ANY_VIS | Emergency Department visits for any cause within the calendar year (counted as ED visit only if not admitted, otherwise counted only as inpatient visit) |
| **PULMONARY FUNCTION TESTING** | |
| SPIRO_YR | spirometry performed within the previous year (yes/no) |
| SPIRO_EVER | spirometry performed ever (since 2001) yes/no |
| **CHARLSON COMORBIDITY INDEX** - BINARY - YES/NO | |
| CC_MET_CA | Charlson comorbidity for metastatic cancer (Yes/No) |
| CC_AIDS_HIV | Charlson comorbidity for HIV/AIDS (Yes/No) |
| CC_CHR_PULM | Charlson comorbidity for Chronic Pulmonary Disease (Yes/No) |
| CC_CONN_TISS_RHEUM | Charlson comorbidity for Connective Tissue Disease/Rheumatologic Disease (Yes/No) |
| CC_MI | Charlson comorbidity for Myocardial Infarction (Yes/No) |
| CC_PLEGIA | Charlson comorbidity for Plegia (Yes/No) |
| CC_CRBRO_VSCLR | Charlson comorbidity for Cerebrovascular Disease (Yes/No) |
| CC_CHF | Charlson comorbidity for Congestive Heart Failure (Yes/No) |
| CC_DEMENTIA | Charlson comorbidity for Dementia (Yes/No) |
| CC_MILD_LIV | Charlson comorbidity for Mild Liver Disease (Yes/No) |
| CC_PERIPH_VSCLR | Charlson comorbidity for Peripheral Vascular Disease (Yes/No) |
| CC_RENAL | Charlson comorbidity for Renal Disease (Yes/No) |
| CC_DM_W_COMP | Charlson comorbidity for Diabetes Mellitus with Complications (Yes/No) |
| CC_MOD_SEV_LIV | Charlson comorbidity for Moderate-Severe Liver Disease (Yes/No) |
| CC_PEPTIC_ULCR | Charlson comorbidity for Peptic Ulcer Disease (Yes/No) |
| CC_CANCER | Charlson comorbidity for Cancer (Yes/No) |
| CC_DM_WO_COMP | Charlson comorbidity for Diabetes Mellitus without complications (Yes/No) |
| **LABORATORY VALUES** | Original data had values and flags. Flags were created using typical normal ranges for these labs in the study time period. Normal ranges sometimes specific by sex or race. |
| MIN_BNP_YR_FLG | Minimum Brain-Natriuretic Peptide within the previous calendar year (high/normal/Low) |
| MAX_BNP_YR_FLG | Maximum Brain-Natriuretic Peptide within the previous calendar year (high/normal/Low) |
| MIN_BNP_EVER_FLG | Minimum Brain-Natriuretic Peptide EVER (high/normal/Low) |
| MAX_BNP_EVER_FLG | Maximum Brain-Natriuretic Peptide within the 10 years (high/normal/Low) |
| MIN_HBA1C_YR_FLG | Minimum Hemoglobin A1C within the previous calendar year (High/normal/low) |
| MAX_HBA1C_YR_FLG | Maximum Hemoglobin A1C within the previous calendar year (High/normal/low) |
| MIN_HBA1C_EVER_FLG | Minimum Hemoglobin A1C EVER (High/normal/low) |
| MAX_HBA1C_EVER_FLG | Maximum Hemoglobin A1C EVER (High/normal/low) |
| MIN_COHB_YR_FLG | Minimum serum Carboxyhemoglobin level within the calendar year (normal/high) |
| MAX_COHB_YR_FLG | Maximum serum Carboxyhemoglobin level within the calendar year (normal/high) |
| MIN_COHB_EVER_FLG | Minimum serum Carboxyhemoglobin level EVER (normal/high) |
| MAX_COHB_EVER_FLG | Maximum serum Carboxyhemoglobin level EVER (normal/high) |
| MIN_PCO2_YR_FLG | Minimum partial pressure of Carbon Dioxide in arterial blood (PaCO2) within the previous calendar year (high/normal/low) |
| MAX_PCO2_YR_FLG | Maximum partial pressure of Carbon Dioxide in arterial blood (PaCO2) within the previous calendar year (high/normal/low) |
| MIN_PCO2_EVER_FLG | Minimum partial pressure of Carbon Dioxide in arterial blood (PaCO2) EVER (high/normal/low) |
| MAX_PCO2_EVER_FLG | Maxiumum partial pressure of Carbon Dioxide in arterial blood (PaCO2) EVER (high/normal/low) |
| **COMPLETE METABOLIC PANEL/CHEMISTRY** | |
| MIN_TOTBILI_YR_FLG | Minimum serum Total bilirubin level within the previous calendar year (high/normal/low) |
| MAX_TOTBILI_YR_FLG | Maxium serum Total bilirubin level within the previous calendar year (high/normal/low) |
| MIN_TOTBILI_EVER_FLG | Minimum serum Total bilirubin level EVER (high/normal/low) |
| MAX_TOTBILI_EVER_FLG | Maxium serum Total bilirubin level EVER (high/normal/low) |
| MIN_ALT_YR_FLG | Minimum serum Alanine Aminotransferase level within the previous calendar year (high/normal/low) |
| MAX_ALT_YR_FLG | Maximum serum Alanine Aminotransferase level within the previous calendar year (high/normal/low) |
| MIN_ALT_EVER_FLG | Minimum serum Alanine Aminotransferase level EVER (high/normal/low) |
| MAX_ALT_EVER_FLG | Maximum serum Alanine Aminotransferase level EVER (high/normal/low) |
| MIN_PROT_YR_FLG | Minimum serum protein level within the calendar year (high/normal/low) |
| MAX_PROT_YR_FLG | Maximum serum protein level within the calendar year (high/normal/low) |
| MIN_PROT_EVER_FLG | Minimum serum protein level EVER (high/normal/low) |
| MAX_PROT_EVER_FLG | Maximum serum protein level EVER (high/normal/low) |
| MIN_BUN_YR_FLG | Minimum serum Blood Urea Nitrogen (BUN) level within the calendar year (high/normal/low) |
| MAX_BUN_YR_FLG | Maximum serum Blood Urea Nitrogen (BUN) level within the calendar year (high/normal/low) |
| MIN_BUN_EVER_FLG | Minimum serum Blood Urea Nitrogen (BUN) level EVER (high/normal/low) |
| MAX_BUN_EVER_FLG | Maximum serum Blood Urea Nitrogen (BUN) level EVER (high/normal/low) |
| MIN_CL_YR_FLG | Minimum serum Chloride level within the calendar year (high/normal/low) |
| MAX_CL_YR_FLG | Maximum serum Chloride level within the calendar year (high/normal/low) |
| MIN_CL_EVER_FLG | Minimum serum Chloride level EVER (high/normal/low) |
| MAX_CL_EVER_FLG | Maximum serum Chloride level EVER (high/normal/low) |
| MAX_K_YR_FLG | Maximum serum Potassium level within the calendar year (high/normal/low) |
| MIN_K_YR_FLG | Minimum serum Potassium level within the calendar year (high/normal/low) |
| MIN_K_EVER_FLG | Minimum serum Potassium level EVER (high/normal/low) |
| MAX_K_EVER_FLG | Maximum serum Potassium level EVER (high/normal/low) |
| MAX_ALB_YR_FLG | Maximum serum Albumin level within the calendar year (high/normal/low) |
| MIN_ALB_EVER_FLG | Minimum serum Albumin level EVER (high/normal/low) |
| MIN_ALB_YR_FLG | Minimum serum Albumin level within the calendar year (high/normal/low) |
| MAX_ALB_EVER_FLG | Maximum serum Albumin level EVER (high/normal/low) |
| MIN_GFR_YR_FLG | Minimum Glomerular Filtration Rate (GFR) within the calendar year (high/normal/low) |
| MAX_GFR_YR_FLG | Maximum Glomerular Filtration Rate (GFR) within the calendar year (high/normal/low) |
| MIN_GFR_EVER_FLG | Minimum Glomerular Filtration Rate (GFR) EVER (high/normal/low) |
| MAX_GFR_EVER_FLG | Maximum Glomerular Filtration Rate (GFR) EVER (high/normal/low) |
| MIN_CREAT_YR_FLG | Minimum serum creatinine level within the calendar year (high/normal/low) |
| MAX_CREAT_YR_FLG | Maximum serum creatinine level within the calendar year (high/normal/low) |
| MIN_CREAT_EVER_FLG | Minimum serum creatinine level EVER (high/normal/low) |
| MAX_CREAT_EVER_FLG | Maximum serum creatinine level EVER (high/normal/low) |
| min_aniongap_ever_flg | Minimum Anion Gap EVER (high/normal/low) |
| max_aniongap_ever_flg | Maximum Anion Gap EVER (high/normal/low) |
| MIN_GLUCOSE_YR_FLG | Minimum Glucose within the calendar year (high/normal/low) |
| MAX_GLUCOSE_YR_FLG | Maximum Glucose within the calendar year (high/normal/low) |
| MIN_GLUCOSE_EVER_FLG | Minimum Glucose EVER (high/normal/low) |
| MAX_GLUCOSE_EVER_FLG | Maximum Glucose EVER (high/normal/low) |
| MIN_NA_YR_FLG | Minimum serum sodium level within the previous calendar year (high/normal/low) |
| MAX_NA_YR_FLG | Maxium serum sodium level within the previous calendar year (high/normal/low) |
| MIN_NA_EVER_FLG | Minimum serum sodium level EVER (high/normal/low) |
| MAX_NA_EVER_FLG | Maximum serum sodium level EVER (high/normal/low) |
| MIN_ALKPHOS_YR_FLG | Minimum serum alkaline phosphatase level within the previous calendar year (high/normal/low) |
| MAX_ALKPHOS_YR_FLG | Maximum serum alkaline phosphatase level within the previous calendar year (high/normal/low) |
| MIN_ALKPHOS_EVER_FLG | Minimum serum alkaline phosphatase level EVER (high/normal/low) |
| MAX_ALKPHOS_EVER_FLG | Maximum serum alkaline phosphatase level EVER (high/normal/low) |
| MIN_CO2_YR_FLG | Minimum serum bicarbonate level within the previous calendar year (high/normal/low) |
| MAX_CO2_YR_FLG | Maximum serum bicarbonate level within the previous calendar year (high/normal/low) |
| MIN_CO2_EVER_FLG | Minimum serum bicarbonate level EVER (high/normal/low) |
| MAX_CO2_EVER_FLG | Maximum serum bicarbonate level EVER (high/normal/low) |
| MIN_AST_YR_FLG | Minimum serum Aspartate Aminotransferase level within the previous calendar year (high/normal/low) |
| MAX_AST_YR_FLG | Maximum serum Aspartate Aminotransferase level within the previous calendar year (high/normal/low) |
| MIN_AST_EVER_FLG | Minimum serum Aspartate Aminotransferase level EVER (high/normal/low) |
| MAX_AST_EVER_FLG | Maximum serum Aspartate Aminotransferase level EVER (high/normal/low) |
| **COMPLETE BLOOD COUNT WITH DIFFERENTIAL** | |
| MIN_PLTS_YR_FLG | Minimum platelet count within the previous calendar year (high/normal/low) |
| MAX_PLTS_YR_FLG | Maximum platelet count within the previous calendar year (high/normal/low) |
| MIN_PLTS_EVER_FLG | Minimum platelet count EVER (high/normal/low) |
| MAX_PLTS_EVER_FLG | Maximum platelet count EVER (high/normal/low) |
| MIN_EOSINCNT_YR_FLG | Minimum peripheral absolute eosinophil count within the previous calendar year (high/normal/low) |
| MAX_EOSINCNT_YR_FLG | Maximum peripheral absolute eosinophil count within the previous calendar year (high/normal/low) |
| MIN_EOSINCNT_EVER_FLG | Minimum peripheral absolute eosinophil count EVER (high/normal/low) |
| MAX_EOSINCNT_EVER_FLG | Maximum peripheral absolute eosinophil count EVER (high/normal/low) |
| MIN_MONOPCT_YR_FLG | Minimum peripheral monocyte percent within the calendar year (high/normal/low) |
| MAX_MONOPCT_YR_FLG | Maximum peripheral monocyte percent within the calendar year (high/normal/low) |
| MIN_MONOPCT_EVER_FLG | Minimum peripheral monocyte percent EVER (high/normal/low) |
| MAX_MONOPCT_EVER_FLG | Maximum peripheral monocyte percent EVER (high/normal/low) |
| MIN_MCH_YR_FLG | Minimum peripheral mean corpuscular hemoglobin level (MCH) within the calendar year (high/normal/low) |
| MAX_MCH_YR_FLG | Maximum peripheral mean corpuscular hemoglobin level (MCH) within the calendar year (high/normal/low) |
| MIN_MCH_EVER_FLG | Minimum peripheral mean corpuscular hemoglobin level (MCH) EVER (high/normal/low) |
| MAX_MCH_EVER_FLG | Maximum peripheral mean corpuscular hemoglobin level (MCH) EVER (high/normal/low) |
| MIN_LYMPHABS_YR_FLG | Minimum peripheral absolute lymphocyte counts within the calendar year (high/normal/low) |
| MAX_LYMPHABS_YR_FLG | Maximum peripheral absolute lymphocyte counts within the calendar year (high/normal/low) |
| MIN_LYMPHABS_EVER_FLG | Minimum peripheral absolute lymphocyte counts EVER (high/normal/low) |
| MAX_LYMPHABS_EVER_FLG | Maximum peripheral absolute lymphocyte counts EVER (high/normal/low) |
| MIN_NEUTABS_YR_FLG | Minimum peripheral absolute neutrophil counts within the calendar year (high/normal/low) |
| MAX_NEUTABS_YR_FLG | Maximum peripheral absolute neutrophil counts within the calendar year (high/normal/low) |
| MIN_NEUTABS_EVER_FLG | Minimum peripheral absolute neutrophil counts EVER (high/normal/low) |
| MAX_NEUTABS_EVER_FLG | Maximum peripheral absolute neutrophil counts EVER (high/normal/low) |
| MIN_HGB_YR_FLG | Minimum serum hemoglobin within the calendar year (high/normal/low) |
| MIN_HGB_EVER_FLG | Maximum serum hemoglobin within the calendar year (high/normal/low) |
| MAX_HGB_YR_FLG | Minimum serum hemoglobin EVER (high/normal/low) |
| MAX_HGB_EVER_FLG | Maximum serum hemoglobin EVER (high/normal/low) |
| MIN_GRANPCT_YR_FLG | Minimum peripheral granulocyte percent within the calendar year (high/normal/low) |
| MAX_GRANPCT_YR_FLG | Maximum peripheral granulocyte percent within the calendar year (high/normal/low) |
| MIN_GRANPCT_EVER_FLG | Minimum peripheral granulocyte percent EVER (high/normal/low) |
| MAX_GRANPCT_EVER_FLG | Maximum peripheral granulocyte percent EVER (high/normal/low) |
| MIN_WBC_YR_FLG | Minimum serum white blood count (WBC) within the calendar year (high/normal/low) |
| MAX_WBC_YR_FLG | Maximum serum White Blood Count (WBC) within the calendar year (high/normal/low) |
| MIN_WBC_EVER_FLG | Minimum serum White Blood Count (WBC) EVER (high/normal/low) |
| MAX_WBC_EVER_FLG | Maximum serum White Blood Count (WBC) EVER (high/normal/low) |
| MIN_MONOABS_YR_FLG | Minimum peripheral absolute monocyte counts within the calendar year (high/normal/low) |
| MAX_MONOABS_YR_FLG | Maximum peripheral absolute monocyte counts within the calendar year (high/normal/low) |
| MIN_MONOABS_EVER_FLG | Minimum peripheral absolute monocyte counts EVER (high/normal/low) |
| MAX_MONOABS_EVER_FLG | Maximum peripheral absolute monocyte counts EVER (high/normal/low) |
| MIN_MCHC_YR_FLG | Minimum peripheral mean corpuscular hemoglobin concentration (MCHC) within the calendar year (high/normal/low) |
| MAX_MCHC_YR_FLG | Maximum peripheral mean corpuscular hemoglobin concentration (MCHC) within the calendar year (high/normal/low) |
| MIN_MCHC_EVER_FLG | Minimum peripheral mean corpuscular hemoglobin concentration (MCHC) EVER (high/normal/low) |
| MAX_MCHC_EVER_FLG | Maximum peripheral mean corpuscular hemoglobin concentration (MCHC) EVER (high/normal/low) |
| MIN_RDW_YR_FLG | Minimum peripheral Red Cell Distribution Width (RDW) within the calendar year (high/normal/low) |
| MAX_RDW_YR_FLG | Maximum peripheral Red Cell Distribution Width (RDW) within the calendar year (high/normal/low) |
| MIN_RDW_EVER_FLG | Minimum peripheral Red Cell Distribution Width (RDW) EVER (high/normal/low) |
| MAX_RDW_EVER_FLG | Maximum peripheral Red Cell Distribution Width EVER (high/normal/low) |
| MIN_RBC_YR_FLG | Minimum peripheral red blood cell counts (RBC) within the calendar year (high/normal/low) |
| MAX_RBC_YR_FLG | Maximum peripheral red blood cell counts (RBC) within the calendar year (high/normal/low) |
| MIN_RBC_EVER_FLG | Minimum peripheral red blood cell counts (RBC) EVER (high/normal/low) |
| MAX_RBC_EVER_FLG | Maximum peripheral red blood cell counts (RBC) EVER (high/normal/low) |
| MIN_MCV_YR_FLG | Minimum peripheral Mean Corpuscular Volume (MCV) within the calendar year (high/normal/low) |
| MAX_MCV_YR_FLG | Maximum peripheral Mean Corpuscular Volume (MCV) within the calendar year (high/normal/low) |
| MIN_MCV_EVER_FLG | Minimum peripheral Mean Corpuscular Volume (MCV) EVER (high/normal/low) |
| MAX_MCV_EVER_FLG | Maximum peripheral Mean Corpuscular Volume (MCV) EVER (high/normal/low) |
| MIN_NEUTPCT_YR_FLG | Minimum peripheral neutrophil percent within the calendar year (high/normal/low) |
| MAX_NEUTPCT_YR_FLG | Maximum peripheral neutrophil percent within the calendar year (high/normal/low) |
| MIN_NEUTPCT_EVER_FLG | Minimum peripheral neutrophil percent EVER (high/normal/low) |
| MAX_NEUTPCT_EVER_FLG | Maximum peripheral neutrophil percent EVER (high/normal/low) |
| MIN_LYMPHPCT_YR_FLG | Minimum peripheral lymphocyte percent within the calendar year (high/normal/low) |
| MAX_LYMPHPCT_YR_FLG | Maximum peripheral lymphocyte percent within the calendar year (high/normal/low) |
| MIN_LYMPHPCT_EVER_FLG | Minimum peripheral lymphocyte percent EVER (high/normal/low) |
| MAX_LYMPHPCT_EVER_FLG | Maximum peripheral lymphocyte percent EVER (high/normal/low) |
| MIN_GRANABS_YR_FLG | Minimum peripheral absolute granulocyte counts within the calendar year (high/normal/low) |
| MAX_GRANABS_YR_FLG | Maximum peripheral absolute granulocyte counts within the calendar year (high/normal/low) |
| MIN_GRANABS_EVER_FLG | Minimum peripheral absolute granulocyte counts EVER (high/normal/low) |
| MAX_GRANABS_EVER_FLG | Maximum peripheral absolute granulocyte counts EVER (high/normal/low) |
| MIN_MPV_YR_FLG | Minimum peripheral Mean Platelet Volume (MPV) within the calendar year (high/normal/low) |
| MAX_MPV_YR_FLG | Maximum peripheral Mean Platelet Volume (MPV) within the calendar year (high/normal/low) |
| MIN_MPV_EVER_FLG | Minimum peripheral Mean Platelet Volume (MPV) EVER (high/normal/low) |
| MAX_MPV_EVER_FLG | Maximum peripheral Mean Platelet Volume (MPV) EVER (high/normal/low) |
| MIN_HCT_YR_FLG | Minimum serum hematocrit within the calendar year (high/normal/low) |
| MAX_HCT_YR_FLG | Maximum serum hematocrit within the calendar year (high/normal/low) |
| MIN_HCT_EVER_FLG | Minimum serum hematocrit EVER (high/normal/low) |
| MAX_HCT_EVER_FLG | Maximum serum hematocrit EVER (high/normal/low) |
| MIN_RDW_SD_YR_FLG | Minimum peripheral Red Cell Distribution Width (RDW) standard deviation within the calendar year (high/normal/low) |
| MAX_RDW_SD_YR_FLG | Maximum peripheral Red Cell Distribution Width (RDW) standard deviation within the calendar year (high/normal/low) |
| MIN_RDW_SD_EVER_FLG | Minimum peripheral Red Cell Distribution Width (RDW) standard deviation EVER (high/normal/low) |
| MAX_RDW_SD_EVER_FLG | Maximum peripheral Red Cell Distribution Width (RDW) standard deviation EVER (high/normal/low) |

**Supplementary Figure 2. Cluster Analysis Dendrogram**

A seven-cluster solution was identified based on the initial goal of identifying four to eight clusters and relative size of each cluster. For example, going from seven to eight clusters did not result in breaking up the largest cluster (Cluster 3), but yielded two smaller clusters from an already small cluster. Thus the seven-cluster solution was selected. This method provided segmentation of the population using clinically similar groupings that were derived independently of study outcome variables.


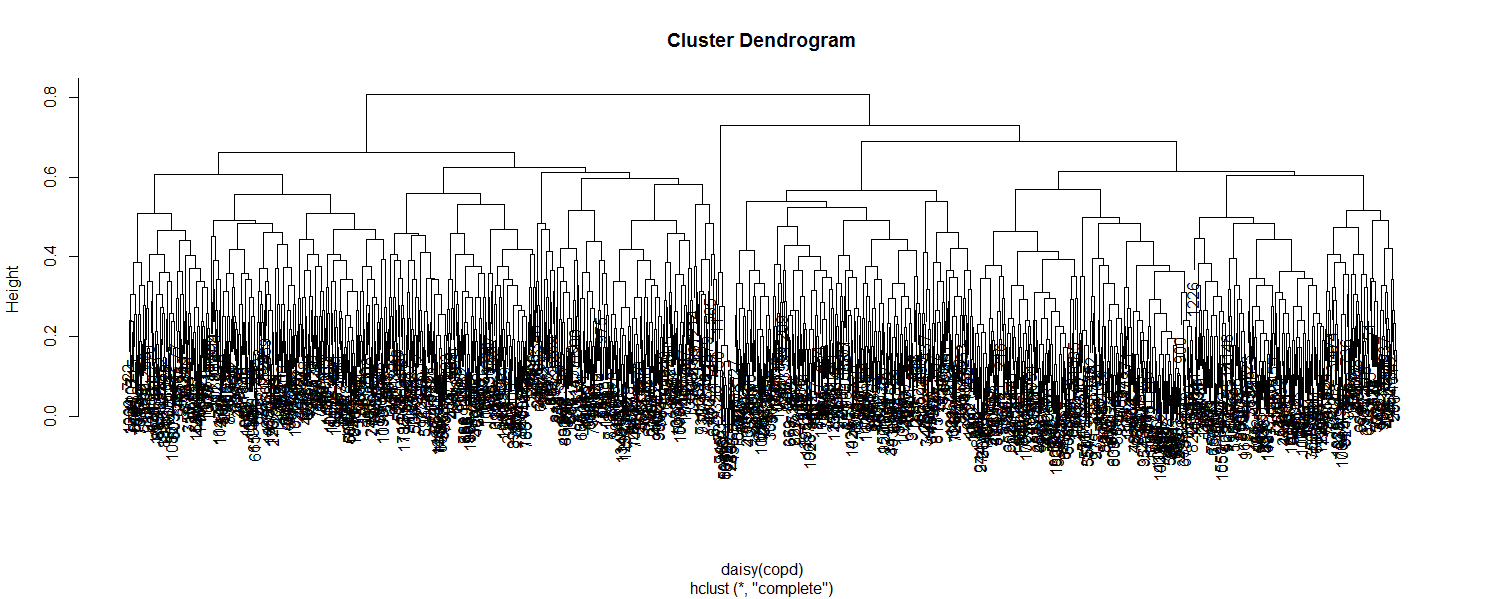


**Supplementary Table 2. Billing Codes for COPD used to define the data sets in the various cohorts**

1. Intermountain Healthcare Derivation Cohort

ICD9 code (491.2, 492) in any sequence or a DRG (190-192) at any inpatient, ED, or ambulatory face-to-face encounter in 2013 or prior were identified.

| 1. Intermountain Healthcare Internal Validation Dataset |
| --- |
| •        J44.0 Chronic obstructive pulmonary disease. |
| •        with acute lower respiratory infection. |
| •        J44.1 Chronic obstructive pulmonary disease. |
| •        with acute exacerbation, unspecified. |
| •        J44.9 Chronic obstructive pulmonary disease, |
| •        unspecified. |
| •        Emphysema, unspecified. **J43.9** |
| •        Chronic bullous emphysema: ICD10: J43.9 |
| •        Diagnosis of COPD: |
| •        COPD: ICD10: J44.1 |
| •        COPD: ICD9: 496 |
| •        Chronic obstructive Lung Disease: ICD9: 496 |
| •        Emphysema: ICD9: 492.* |
| •        Chronic bronchitis: ICD9: 491.9; ICD10 J42 |
| •        Obstructive chronic bronchitis: ICD9: 491.21 |
| •        Pulmonary emphysema: ICD9: 492.8 |
| •        Pulmonary emphysema: ICD10: J43.0 |
| •        Chronic airway obstruction: ICD9: 496 |
| •        Chronic bullous emphysema: ICD10: J43.9 |
| •        Chronic bullous emphysema: ICD9: 429.0 |

1. National Veterans Affairs Health System Validation Dataset

Diagnosis of COPD:

- 1. COPD: ICD10: J44.1
  2. COPD: ICD9: 496
  3. Chronic obstructive Lung Disease: ICD9: 496
  4. Emphysema: ICD9: 492.*
  5. Chronic bronchitis: ICD9: 491.9
  6. Obstructive chronic bronchitis: ICD9: 491.21
  7. Pulmonary emphysema: ICD9: 492.8
  8. Pulmonary emphysema: ICD10: J43.0
  9. Chronic airway obstruction: ICD9: 496
  10. Chronic bullous emphysema: ICD10: J43.9
  11. Chronic bullous emphysema: ICD9: 429.0

1. University of Chicago Medicine System Validation Dataset

ICD9 code 491.2 or 492.x.

**Supplementary Figure 3. Consort Diagram of the Internal Validation Cohort at Intermountain Healthcare**

**
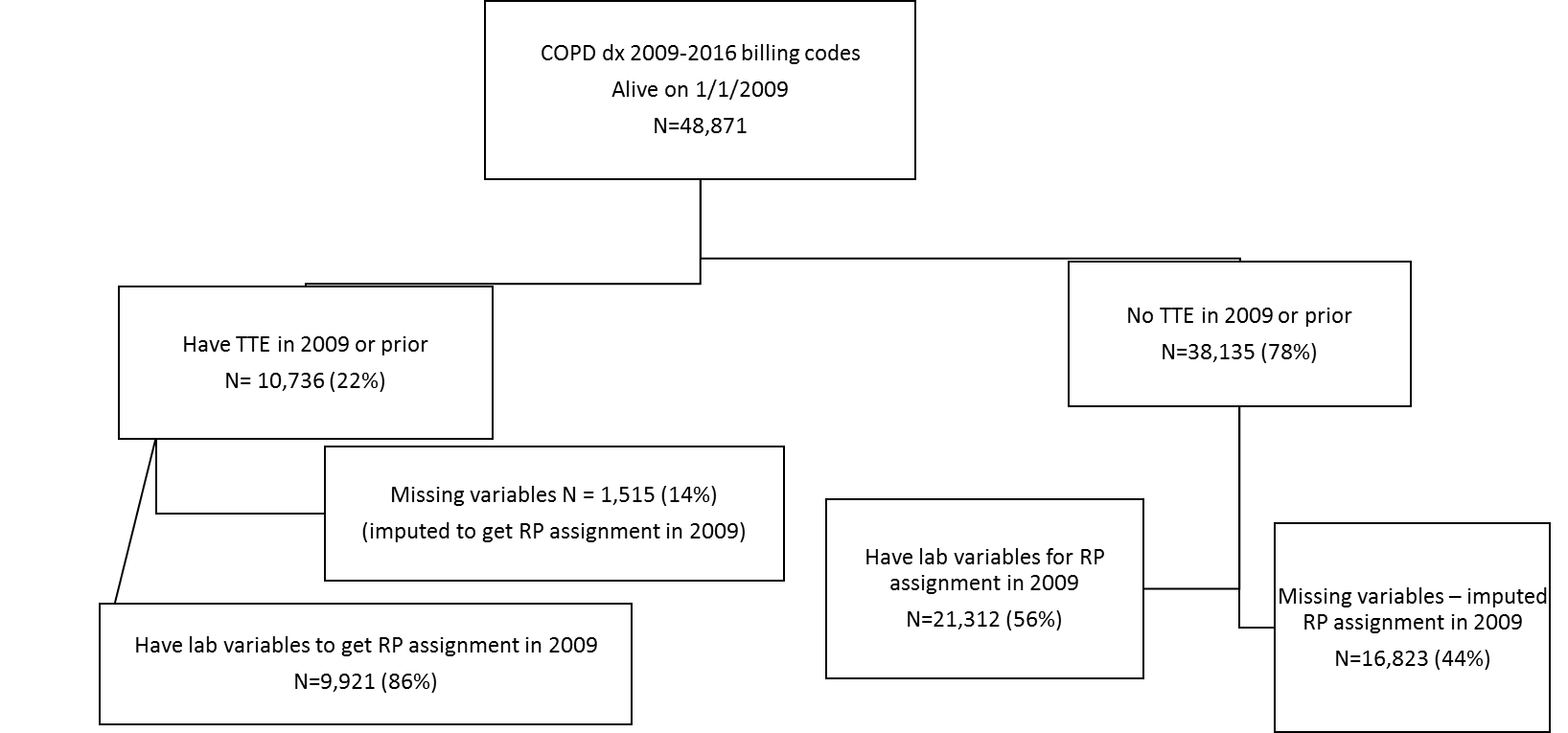
**

**Supplementary Figure 4. Consort Diagram of the External Validation Cohort at the National Veterans Affairs Health System**

**
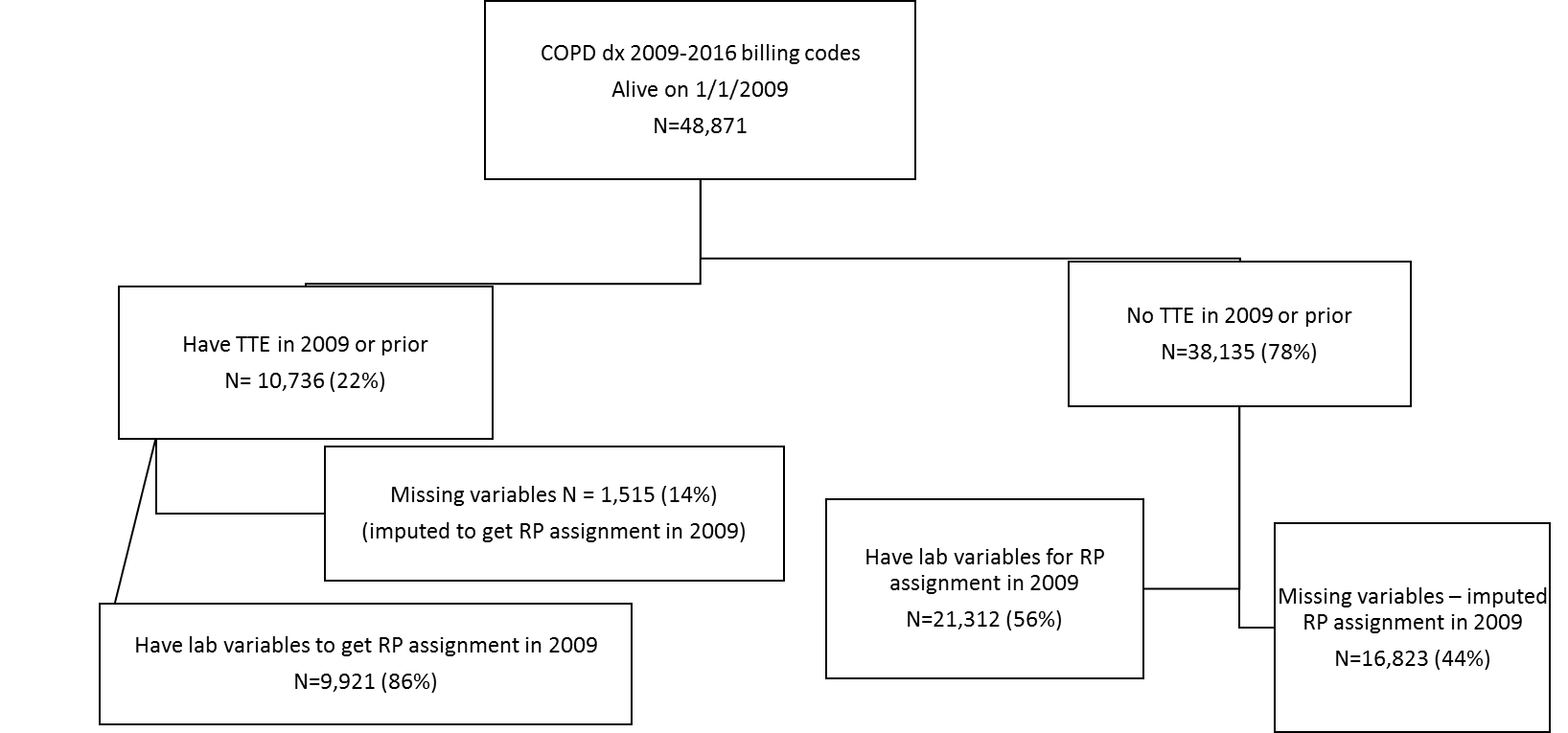
**

**Supplementary Figure 5. Consort Diagram of the External Validation Cohort at the University of Chicago Medicine System**

**
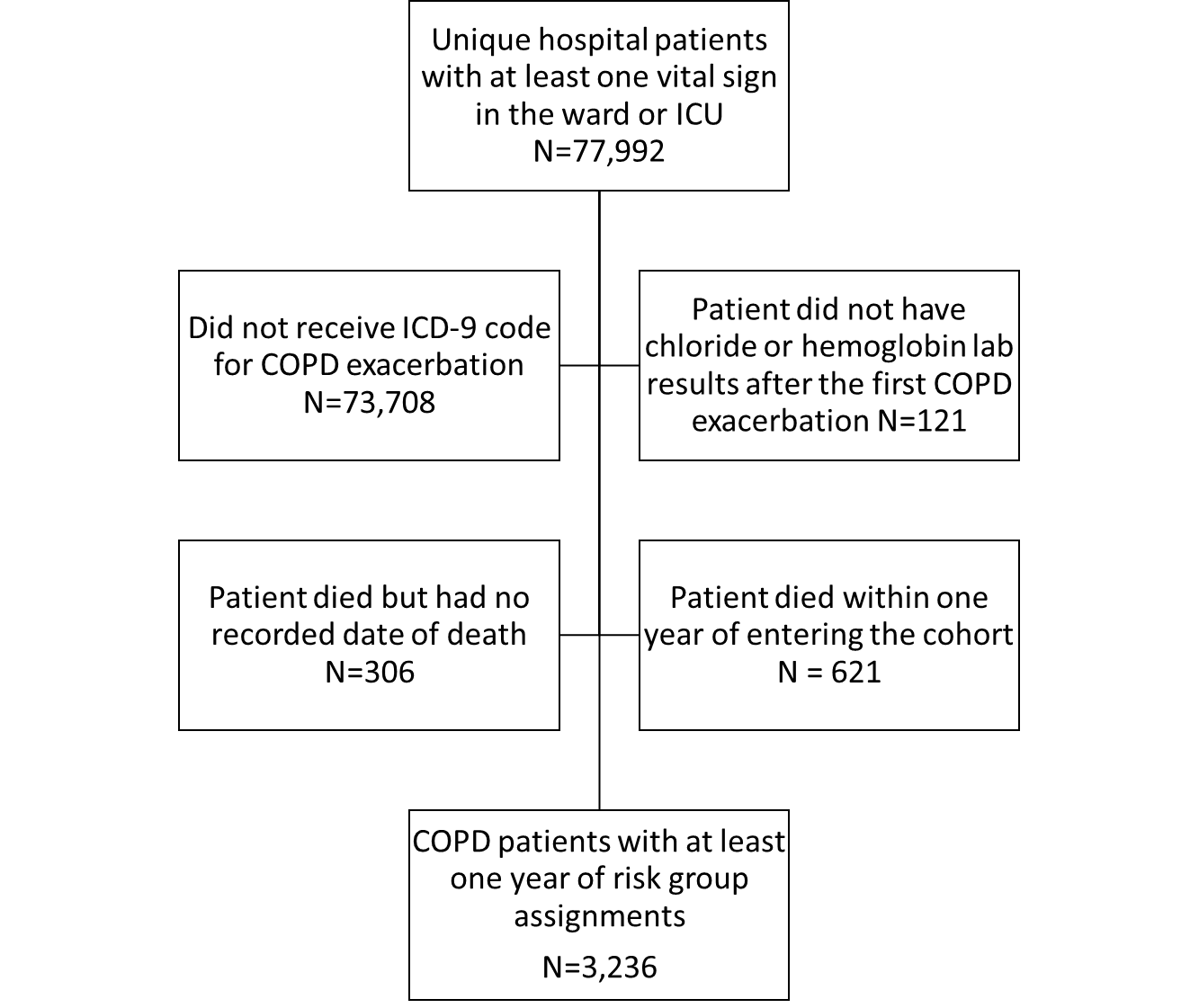
**

**Supplementary Figure 6. PFT data in Derivation Cohort**

Panel A. Consort Diagram of PFT data availability in Derivation Cohort. Encoded PFT data were available in minority of patients.

Panel B. PFT interpretations in the 535 patients with encoded PFT data of the 5006 Cluster Cohort


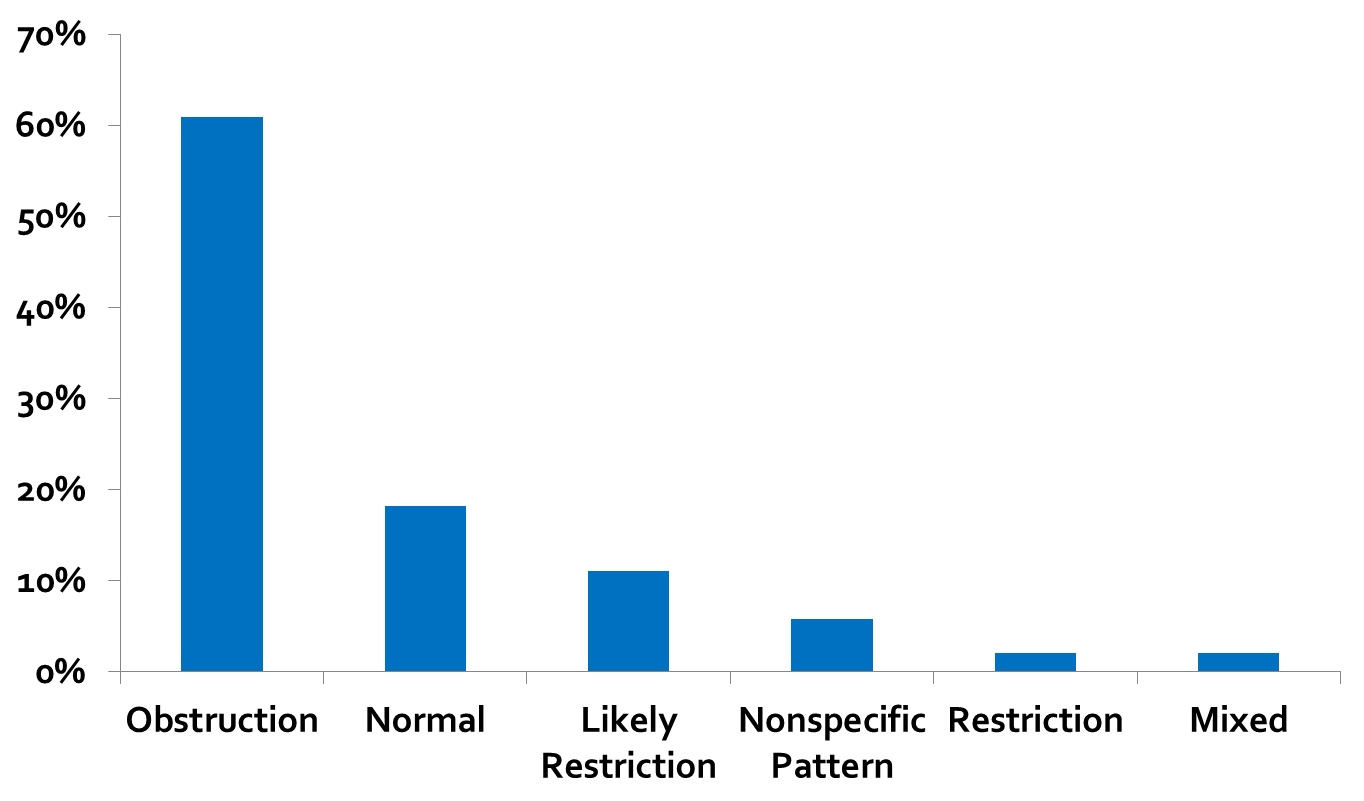


**Supplementary Table 3. Demographics and Clinical Characteristics of N=5006 Patients in 2013**

| **Variable Name** | **Total** | **Cluster 1** | **Cluster 5** | **Cluster 4** | **Cluster 6** | **Cluster 3** | **Cluster 2** | **Cluster 7** | **P value** |
| --- | --- | --- | --- | --- | --- | --- | --- | --- | --- |
| **Number of patients, N (%)** | 5006 | 524 (11) | 774 (16) | 1217 (24) | 251 (5) | 1499 (30) | 662 (13) | 79 (2) | <0.001 |
| **Age, mean (SD)** | 70 (13) | 72 (11) | 64 (13) | 69 (12) | 69 (12) | 75 (11) | 66 (12) | 71 (12) | <0.001 |
| **Female, N (%)** | 2416 (48) | 220 (42) | 407 (53) | 624 (51) | 108 (43) | 640 (43) | 387 (59) | 30 (38) | <0.001 |
| **White, N (%)** | 4669 (93) | 491 (94) | 710 (92) | 1139 (94) | 234 (93) | 1398 (93) | 626 (95) | 71 (90) | 0.371 |
| **4-yr mortality N (%)** | 1432 (29) | 294 (56) | 59 (8) | 209 (17) | 61 (24) | 521 (35) | 269 (41) | 19 (24) | <0.001 |
| **Pulmonary Function Testing** |  |  |  |  |  |  |  |  |  |
| **Number of PFT cases, N (%)** | 535 (11) | 40 (8) | 95 (12) | 132 (11) | 25 (10) | 163 (11) | 76 (12) | 4 (5) | 0.107 |
| **FEV1 (L), mean (SD)** | 2.9 (0.7) | 2.8 (0.8) | 3.1 (0.7) | 2.9 (0.7) | 2.9 (0.6) | 2.8 (0.7) | 2.8 (0.7) | 3.2 (0.4) | 0.095 |
| **FEV1 % predicted, mean (SD)** | 63 (22) | 56 (19) | 68 (24) | 65 (23) | 70 (25) | 63 (22) | 59 (22) | 53 (8) | 0.021 |
| **FVC (L), mean (SD)** | 3.8 (0.9) | 3.8 (1.0) | 4.0 (0.8) | 3.8 (0.9) | 3.8 (0.8) | 3.7 (0.9) | 3.6 (0.9) | 4.2 (0.5) | 0.208 |
| **FVC % predicted, mean (SD)** | 82 (20) | 72 (19) | 88 (18) | 84 (17) | 86 (23) | 80 (20) | 77 (21) | 74 (12) | <0.001 |
| **FEV1/FVC, mean (SD)** | 0.59 (0.2) | 0.60 (0.2) | 0.59 (0.2) | 0.59 (0.2) | 0.62 (0.2) | 0.59 (0.1) | 0.59 (0.2) | 0.56 (0.1) | 0.975 |
| **Healthcare Utilization** |  |  |  |  |  |  |  |  |  |
| **ED & Inpatient COPD visits/year, mean (SD)** | 0.34 (0.7) | 0.69 (0.9) | 0.12 (0.3) | 0.23 (0.5) | 0.35 (0.6) | 0.24 (.5) | 0.76 (1.1) | 0.27 (0.5) | <0.001 |
| **ED COPD visits/year, mean (SD)** | 0.09 (0.4) | 0.11 (0.4) | 0.07 (0.3) | 0.10 (0.4) | 0.06 (0.3) | 0.06 (0.3) | 0.19 (0.5) | 0.04 (0.2) | <0.001 |
| **Inpatient COPD visits/year, mean (SD)** | 0.24 (0.5) | 0.58 (0.8) | 0.02 (0.2) | 0.13 (0.4) | 0.29 (0.5) | 0.18 (0.4) | 0.57 (0.9) | 0.23 (0.4) | <0.001 |
| **Outpatient COPD visits/year, mean (SD)** | 0.21 (0.6) | 0.16 (0.6) | 0.21 (0.5) | 0.26 (0.6) | 0.11 (0.4) | 0.19 (0.6) | 0.23 (0.7) | 0.04 (0.2) | <0.001 |
| **ED & Inpatient Any Cause Visits/year , mean (SD)** | 1.35 (1.8) | 2.65 (2.1) | 0.58 (1.0) | 0.98 (1.4) | 1.06 (1.4) | 1.08 (1.5) | 2.68 (2.3) | 0.84 (1.1) | <0.001 |
| **ED any cause visits/year, mean (SD)** | 0.69 (1.1) | 0.96 (1.3) | 0.44 (0.9) | 0.65 (1.1) | 0.48 (1.0) | 0.57 (1.0) | 1.22 (1.4) | 0.19 (0.4) | <0.001 |
| **Inpatient any cause visits/year, mean (SD)** | 0.66 (1.0) | 1.68 (1.3) | 0.14 (0.4) | 0.33 (0.6) | 0.59 (0.8) | 0.50 (0.8) | 1.46 (1.4) | 0.65 (1.0) | <0.001 |
| **Outpatient any cause visits/year, mean (SD)** | 2.18 (1.8) | 2.19 (1.8) | 2.04 (1.7) | 2.38 (1.7) | 1.57 (1.7) | 2.11 (1.8) | 2.51 (1.7) | 0.80 (1.4) | <0.001 |

**Supplementary Table 4. Comorbidities and Lab Results for N=5006 Patients in 2013**

| **Variable Name, N (%)** | **Total** | **Cluster 1** | **Cluster 5** | **Cluster 4** | **Cluster 6** | **Cluster 3** | **Cluster 2** | **Cluster 7** | **P value** |
| --- | --- | --- | --- | --- | --- | --- | --- | --- | --- |
| **Number of patients** | 5006 | 524 (11) | 774 (16) | 1217 (24) | 251 (5) | 1499 (30) | 662 (13) | 79 (2) | <0.001 |
| **Charlson Comorbidity Rate** |  |  |  |  |  |  |  |  |  |
| **Malignancy** | 1135 (23) | 158 (30) | 97 (13) | 227 (19) | 79 (32) | 390 (26) | 173 (26) | 11 (14) | <0.001 |
| **Diabetes** | 2231 (45) | 324 (62) | 157 (20) | 505 (42) | 71 (28) | 791 (53) | 356 (54) | 27 (34) | <0.001 |
| **Liver Disease** | 1293 (26) | 181 (35) | 122 (16) | 279 (23) | 62 (25) | 323 (22) | 305 (46) | 21 (27) | <0.001 |
| **Chronic Pulm. Disease** | 4991 (98) | 519 (99) | 740 (96) | 1197 (98) | 245 (98) | 1475 (98) | 658 (99) | 77 (98) | <0.001 |
| **Rheumat. Disease** | 566 (11) | 80 (15) | 48 (6) | 147 (12) | 16 (6) | 157 (11) | 112 (17) | 6 (8) | <0.001 |
| **Myocardial Infarction** | 1511 (30) | 195 (37) | 121 (16) | 294 (24) | 49 (20) | 597 (40) | 224 (34) | 31 (39) | <0.001 |
| **Cerebrovas. Disease** | 1671 (33) | 214 (41) | 163 (21) | 390 (32) | 69 (28) | 588 (39) | 237 (36) | 10 (13) | <0.001 |
| **Chronic Heart Failure** | 2883 (58) | 433 (83) | 192 (25) | 576 (47) | 105 (42) | 1087 (73) | 432 (65) | 58 (73) | <0.001 |
| **Dementia** | 225 (5) | 32 (6) | 8 (1) | 47 (4) | 7 (3) | 92 (6) | 34 (5) | 5 (6) | <0.001 |
| **Periph. Vasc. Disease** | 1973 (39) | 282 (54) | 137 (18) | 446 (37) | 63 (25) | 742 (50) | 276 (42) | 27 (34) | <0.001 |
| **Renal Disease** | 1491 (30) | 311 (59) | 29 (4) | 161 (13) | 29 (12) | 711 (47) | 219 (33) | 31 (39) | <0.001 |
| **Peptic Ulcer Disease** | 1511 (30) | 195 (37) | 121 (16) | 294 (24) | 49 (20) | 597 (40) | 224 (34) | 31 (39) | <0.001 |
| **Laboratory Value Abnormalities** |  |  |  |  |  |  |  |  |  |
| **Max BNP Ever High** | 2794/3835 (73) | 437/486 (90) | 146/376 (39) | 518/885 (59) | 116/164 (71) | 1066/1279 (83) | 451/584 (77) | 59/61  (97) | <0.001 |
| **Max HBA1C Ever High** | 1779/3041 (59) | 237/376 (63) | 120/300 (40) | 406/720 (56) | 48/126 (38) | 660/987 (67) | 288/491 (59) | 20/41  (49) | <0.001 |
| **Max PCO2 Ever High** | 2431/3266 (74) | 395/450 (88) | 120/260 (46) | 491/701 (70) | 106/153 (69) | 818/1071 (76) | 458/570 (80) | 43/61  (71) | <0.001 |
| **Max CO2 Ever High** | 3224/4984 (65) | 443/524 (85) | 206/763 (27) | 743/1208 (62) | 145/249 (58) | 1082/1499 (72) | 561/662 (85) | 44/79  (56) | <0.001 |
| **Max eosinophil count Ever High** | 1314/4922 (27) | 194/524 (37) | 84/738 (11) | 257/1191 (22) | 42/245 (17) | 452/1489 (30) | 276/661 (42) | 9/74  (12) | <0.001 |
| **Max Albumin Ever High** | 526/4944 (11) | 39/524  (7) | 57/740  (8) | 152/1197 (13) | 23/244  (9) | 113/1498 (8) | 137/662 (21) | 5/79  (6) | <0.001 |
| **Max Creatinine Ever High** | 2764/4987 (55) | 455/524 (87) | 104/765 (14) | 511/1209 (42) | 89/249 (36) | 1143/1499 (76) | 404/662 (61) | 58/79  (73) | <0.001 |
| **Min HGB Ever Low** | 1002/4992  (20) | 3/524  (0.6) | 520/768  (68) | 333/1211  (28) | 43/249  (17) | 82/1499  (6) | 16/662  (2) | 5/79  (6) | <0.001 |

**Supplementary Figure 7. Preferred RP Derived Decision Tree**

The RP tree assigns each patient to a LIVE Score based on the variables in the tree. The concordance between the LIVE Score assignment and the original Cluster assignment is visible in the graphs within each group. For example, the majority of patients assigned in LIVE Score 2 by the tree were in Cluster 2, however, as can be seen by the histogram, there is a significant minority of patients from Cluster 1, who are assigned to LIVE Score 2 by the Decision tree.

**
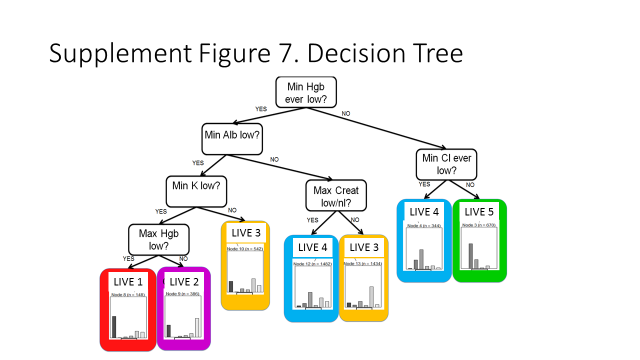
**

**Supplementary Figure 8. Correlation between cluster assignment and RP tree LIVE Score assignment for patients with no missing data.**

The correlation between the RP assigned LIVE Score and the original Cluster assignment is shown for all 3898 patients without missing data that allow RP tree assignment. The Cluster number is on the vertical axis while the LIVE Score number is on the horizontal axis. Along the diagonal axis shown by the large circles representing the majority of patients, are the patients who are concordant between the two assignments. Patients off the diagonal (smaller numbers indicated by smaller circles) show patients where a discrepancy between the LIVE Score assignment and the Cluster type is present.


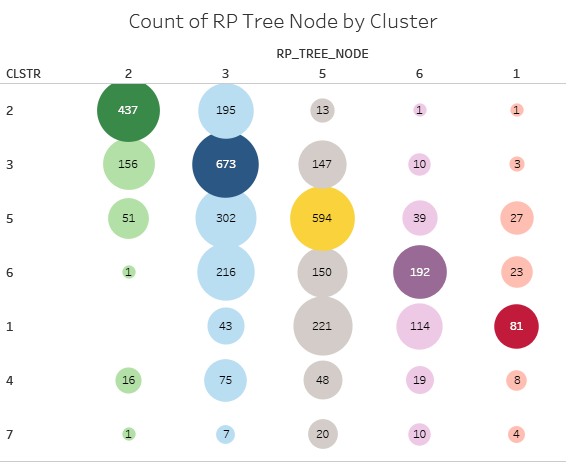


5

5

4

4

3

2

2

6

3

**Supplementary Figure 9. Correlation between cluster assignment and RP tree LIVE Score assignment for patients with no missing data.**

The correlation between the RP assigned LIVE Score and the original Cluster assignment is shown for the 1108 patients with missing data, which were imputed normal to allow RP tree LIVE Score assignment. The Cluster number is on the vertical axis while the LIVE Score number is on the horizontal axis. Along the diagonal axis shown by the large circles representing the majority of patients, are the patients who are concordant between the two assignments. Patients off the diagonal (smaller numbers indicated by smaller circles) show patients where a discrepancy between the LIVE Score assignment and the Cluster type is present.


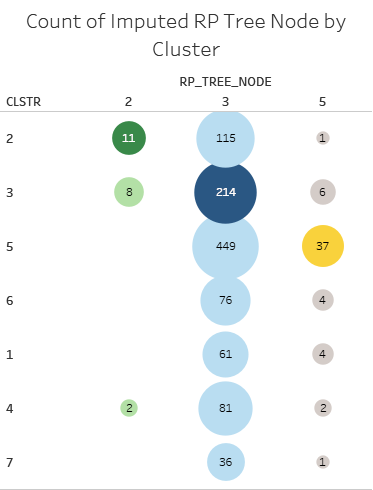


5

4

3

2

1

6

7

5 4 3

**Supplementary Table 5. Demographics and Clinical Characteristics for 38135 Patients without Prior TTE and no data imputing in 2009**

| **Variable Name** | **Total** | **LIVE 1** | **LIVE 5** | **LIVE 4** | **LIVE 3** | **LIVE 2** | **P value** |
| --- | --- | --- | --- | --- | --- | --- | --- |
| **Number of patients, N (%)** | 38135 | 124 (0.3) | 12742 (33) | 5749 (15) | 2204 (6) | 493 (1) | <0.001 |
| **Age, mean (SD)** | 60 (15) | 69 (15) | 58 (15) | 63 (15) | 69 (14) | 61 (15) | <0.001 |
| **Female, N (%)** | 11242 (53) | 40 (32) | 6657 (52) | 3190 (56) | 1046 (48) | 309 (63) | <0.001 |
| **White, N (%)** | 19482 (92) | 101 (82) | 11738 (92) | 5234 (91) | 1972 (90) | 437 (89) | <0.001 |
| **8-yr mortality N (%)** | 4836 (23) | 70 (57) | 1888 (15) | 1664 (29) | 1006 (46) | 208 (42) | <0.001 |
| **Healthcare Utilization** |  |  |  |  |  |  |  |
| **ED & Inpatient COPD visits/year, mean (SD)** | 0.22 (0.7) | 1.01 (1.1) | 0.10 (0.4) | 0.28 (0.8) | 0.52 (1.1) | 1.04 (1.2) | <0.001 |
| **ED COPD visits/year, mean (SD)** | 0.10 (0.5) | 0.26 (0.7) | 0.06 (0.3) | 0.14 (0.6) | 0.18 (0.7) | 0.21 (0.7) | <0.001 |
| **Inpatient COPD visits/year, mean (SD)** | 0.11 (0.4) | 0.75 (0.7) | 0.03 (0.2) | 0.13 (0.4) | 0.34 (0.7) | 0.83 (0.9) | <0.001 |
| **Outpatient COPD visits/year, mean (SD)** | 0.32 (1.0) | 0.13 (0.4) | 0.26 (0.8) | 0.42 (1.2) | 0.41 (1.2) | 0.26 (0.9) | <0.001 |
| **ED & Inpatient Any Cause Visits/year , mean (SD)** | 0.84 (1.8) | 2.52 (3) | 0.49 (1.2) | 1.12 (2.1) | 1.57 (2.4) | 3.19 (2.9) | <0.001 |
| **ED any cause visits/year, mean (SD)** | 0.61 (1.6) | 1.12 (2.4) | 0.42 (1.2) | 0.83 (1.9) | 0.89 (2.1) | 1.43 (2.3) | <0.001 |
| **Inpatient any cause visits/year, mean (SD)** | 0.24 (0.6) | 1.40 (1.0) | 0.07 (0.3) | 0.29 (0.6) | 0.67 (0.9) | 1.77 (1.4) | <0.001 |
| **Outpatient any cause visits/year, mean (SD)** | 2.98 (4.1) | 2.30 (3.6) | 2.33 (3.4) | 3.85 (4.6) | 4.26 (5.1) | 4.04 (5.2) | <0.001 |

**Supplementary Table 6. Comorbidities and Lab Results for 38135 Patients without prior TTE and no data imputing in 2009**

| **Variable Name, N (%)** | **Total** | **LIVE 1** | **LIVE 5** | **LIVE 4** | **LIVE 3** | **LIVE 2** | **P value** |
| --- | --- | --- | --- | --- | --- | --- | --- |
| **Number of patients** | 38135 | 124 (0.3) | 12742 (33) | 5749 (15) | 2204 (6) | 493 (1) | <0.001 |
| **Charlson Comorbidity Rate** |  |  |  |  |  |  |  |
| **Malignancy** | 2610 (12) | 42 (34) | 967 (8) | 941 (16) | 553 (25) | 107 (22) | <0.001 |
| **Diabetes** | 5347 (25) | 42 (34) | 2279 (18) | 1931 (34) | 940 (43) | 155 (31) | <0.001 |
| **Liver Disease** | 2833 (13) | 27 (22) | 1241 (10) | 1018 (18) | 420 (19) | 127 (26) | <0.001 |
| **Chronic Pulm. Disease** | 14914 (70) | 102 (82) | 8107 (64) | 4471 (78) | 1805 (82) | 429 (87) | <0.001 |
| **Rheumat. Disease** | 1499 (7) | 10 (8) | 611 (5) | 577 (10) | 252 (11) | 49 (10) | <0.001 |
| **Myocardial Infarction** | 1842 (9) | 20 (16) | 644 (5) | 687 (12) | 436 (20) | 55 (11) | <0.001 |
| **Cerebrovas. Disease** | 2549 (12) | 28 (23) | 967 (8) | 942 (16) | 523 (24) | 89 (18) | <0.001 |
| **Chronic Heart Failure** | 3099 (15) | 35 (28) | 936 (7) | 1202 (21) | 819 (37) | 107 (22) | <0.001 |
| **Dementia** | 340 (2) | 10 (8) | 86 (0.7) | 134 (2) | 88 (4) | 22 (5) | <0.001 |
| **Periph. Vasc. Disease** | 2976 (14) | 32 (26) | 1077 (9) | 1114 (19) | 662 (30) | 91 (19) | <0.001 |
| **Renal Disease** | 1551 (7) | 34 (27) | 294 (2) | 337 (6) | 806 (37) | 80 (16) | <0.001 |
| **Peptic Ulcer Disease** | 1790 (8) | 20 (16) | 663 (5) | 685 (12) | 333 (15) | 89 (18) | <0.001 |
| **Laboratory Value Abnormalities** |  |  |  |  |  |  |  |
| **Max BNP Ever High** | 968/1891 (51) | 39/44 (89) | 174/518 (34) | 289/645 (45) | 355/529 (67) | 111/155 (72) | <0.001 |
| **Max HBA1C Ever High** | 3858/6374 (61) | 25/43 (58) | 1531/2745 (56) | 1489/2342 (64) | 711/1043 (68) | 102/201 (51) | <0.001 |
| **Max PCO2 Ever High** | 1896/3392 (56) | 28/49 (57) | 404/986 (41) | 854/1354 (63) | 440/731 (60) | 170/272 (63) | <0.001 |
| **Max CO2 Ever High** | 5069/21273 (24) | 59/124 (48) | 1442/12705 (11) | 2303/5748 (40) | 969/2203 (44) | 296/493 (60) | <0.001 |
| **Max eosinophil count Ever High** | 2529/20129 (13) | 32/121 (26) | 939/11745 (8) | 910/5612 (16) | 514/2164 (24) | 134/487 (28) | <0.001 |
| **Max Albumin Ever High** | 1613/20219 (8) | 5/124 (4) | 786/11707 (7) | 598/5691 (11) | 155/2204 (7) | 69/493 (14) | <0.001 |
| **Max Creatinine Ever High** | 5476/21300 (26) | 69/124 (56) | 1773/12733 (14) | 1578/5746 (28) | 1809/2204 (82) | 247/493 (50) | <0.001 |
| **Min HGB Ever Low** | 6816/21312 (32) | 124/124 (100) | 0/12742 (0) | 3995/5749 (70) | 2204/2204 (100) | 493/493 (100) | <0.001 |

**Supplementary Figure 10. Kaplan-Meier survival curve for 1515 patients with a TTE in 2009 or prior and missing variables imputed as normal**

Figure 9 shows the survival spread between the three LIVE Scores with the same pattern. LIVE Score 5 has the lowest overall 8 year mortality and LIVE Score 3 has the highest mortality of the three LIVE Scores assigned in this cohort (LIVE Scores 5, 4, and 3). LIVE Scores 1 and 2 were not assigned in these cohorts where missing variables were imputed as normal, because abnormal values are needed in the tree to result in assignment to LIVE Score 1 or 2.


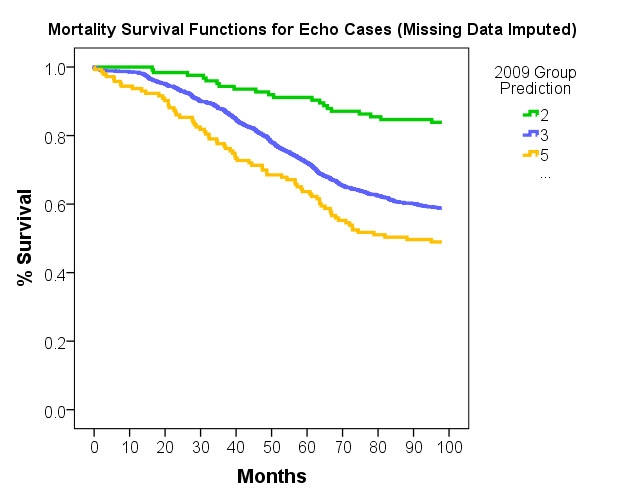


Live Score 2009

5

4

3

**Supplementary Figure 11. Kaplan-Meier survival curve for 16823 patients with no prior TTE in 2009 and missing variables imputed as normal**

Figure 10 shows the survival spread between the three LIVE Scores with the same pattern. LIVE Score 5 has the lowest overall 8 year mortality and LIVE Score 3 has the highest mortality of the three LIVE Scores assigned in this cohort ( LIVE Scores 3,4, and 5). LIVE Scores 1 and 2 were not assigned in these cohorts where missing variables were imputed as normal, because abnormal values are needed in the tree to result in assignment to LIVE Score 1 or 2.


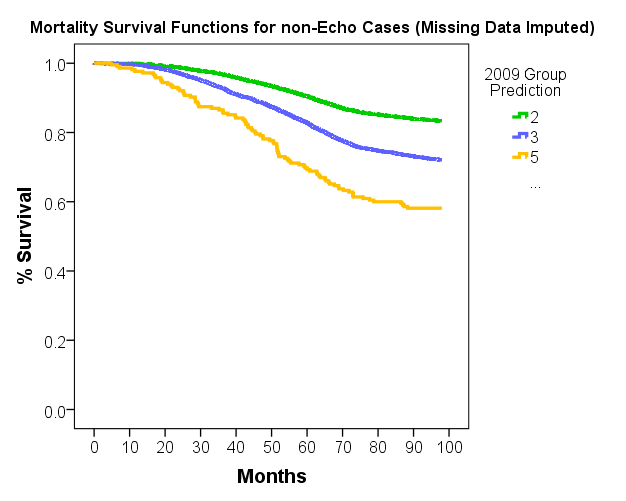


5

4

3

Live Score 2009

**Supplementary Figure 12. Kaplan-Meier survival curve for VA patients based on whether or not they had a TTE in 2009 or prior**

**
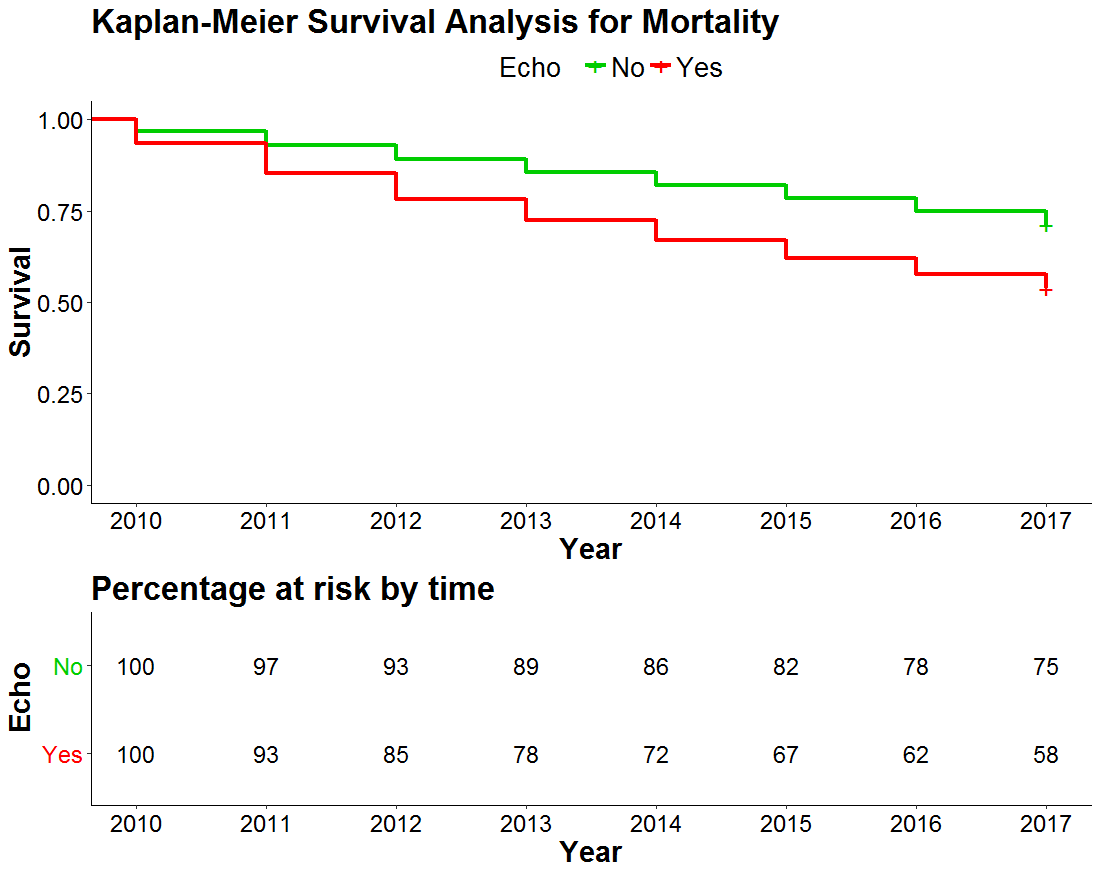
**

**Supplementary Figure 13. Kaplan-Meier survival curve for first COPD exacerbation in VA patients with a TTE in 2009 or prior**

**
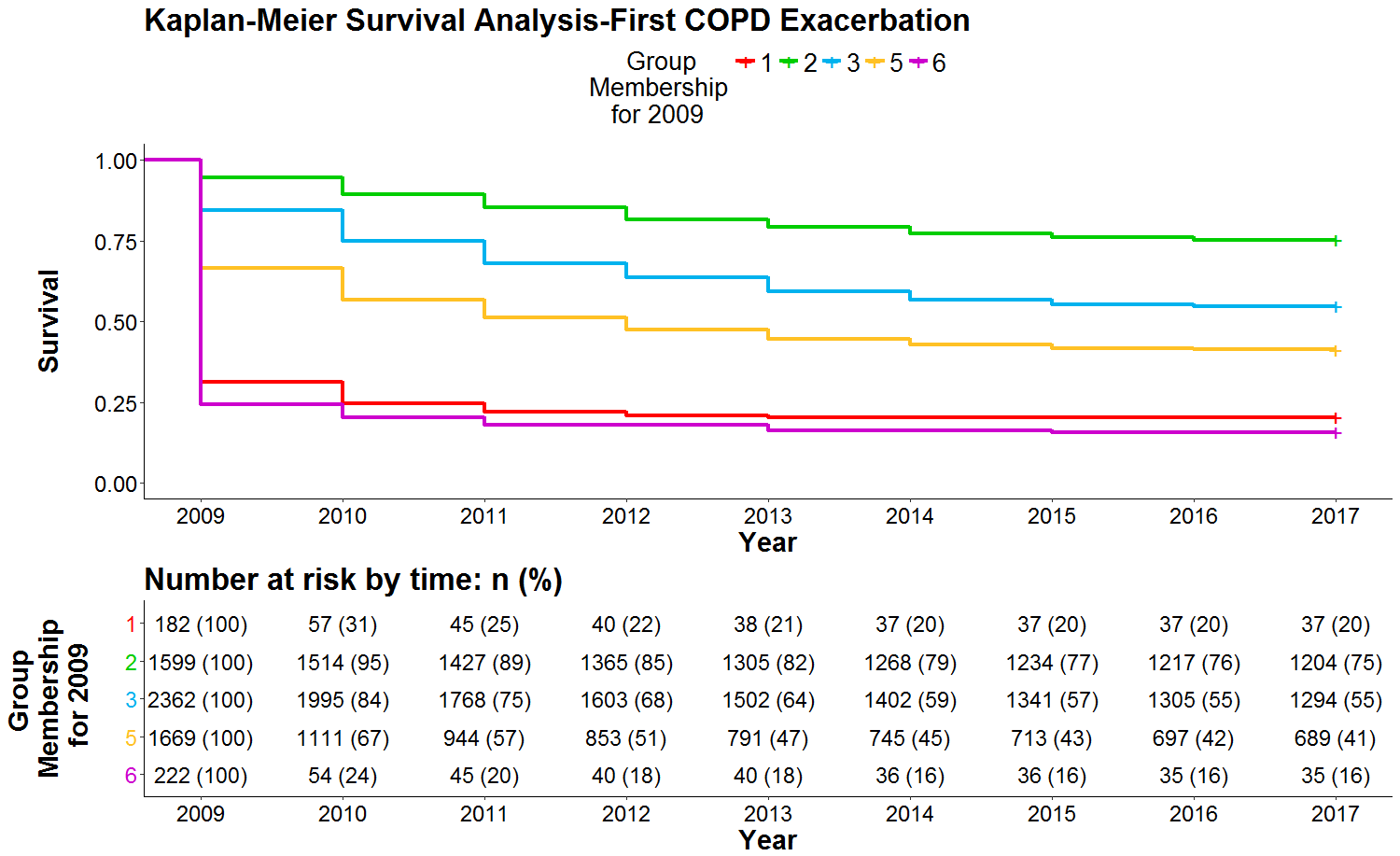
**

| **Comparison b/w Groups** | **P-value** |
| --- | --- |
| 5 vs 4 | **<0.001** |
| 4 vs 3 | **<0.001** |
| 3 vs 2 | **<0.001** |
| 2 vs 1 | 0.208 |
| Overall | **<0.001** |

**Supplementary Figure 14. Kaplan-Meier survival curve for first COPD exacerbation in VA patients without a TTE in 2009 or prior**

**Supplementary Figure 14. Kaplan-Meier survival curve for first COPD exacerbation in VA patients without a TTE in 2009 or prior**

**
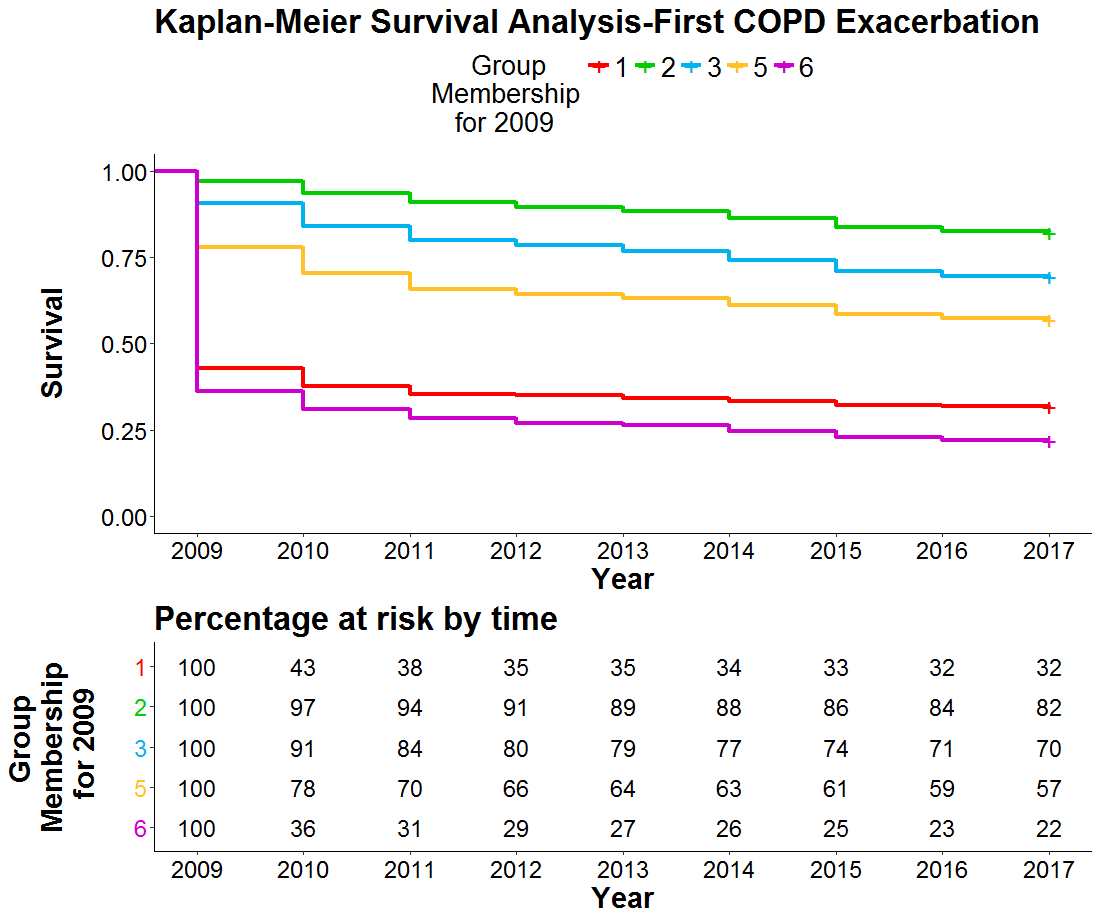
**

| **Comparison b/w Groups** | **P-value** |
| --- | --- |
| **5 vs 4** | **<0.001** |
| **4 vs 3** | **<0.001** |
| **3 vs 2** | **<0.001** |
| **2 vs 1** | **<0.001** |
| **Overall** | **<0.001** |

**Supplementary Table 7. Distribution of the patients by state in the external validation cohort from the Veterans Affairs (VA) informatics and computing infrastructure (VINCI).**

<http://www.hsrd.research.va.gov/for_researchers/vinci/>

|  | **Group** | | | | |
| --- | --- | --- | --- | --- | --- |
| **States** | **1** | **2** | **3** | **5** | **6** |
| *Missing* | 1 | 7 | 14 | 14 | 1 |
| ALABAMA | 0 | 13 | 34 | 17 | 1 |
| ALASKA | 1 | 1 | 1 | 0 | 0 |
| ARIZONA | 3 | 41 | 41 | 28 | 5 |
| ARKANSAS | 0 | 14 | 21 | 4 | 0 |
| CALIFORNIA | 46 | 231 | 350 | 325 | 48 |
| COLORADO | 0 | 6 | 3 | 0 | 0 |
| CONNECTICUT | 4 | 12 | 4 | 7 | 1 |
| DELAWARE | 0 | 1 | 0 | 0 | 0 |
| DISTRICT OF COLUMBIA | 0 | 1 | 3 | 8 | 1 |
| FLORIDA | 12 | 109 | 133 | 79 | 17 |
| GEORGIA | 0 | 10 | 17 | 12 | 2 |
| HAWAII | 0 | 0 | 1 | 0 | 0 |
| IDAHO | 0 | 9 | 11 | 4 | 1 |
| ILLINOIS | 5 | 40 | 31 | 31 | 4 |
| INDIANA | 0 | 18 | 38 | 13 | 2 |
| IOWA | 0 | 28 | 18 | 14 | 4 |
| KANSAS | 0 | 39 | 48 | 26 | 8 |
| KENTUCKY | 4 | 43 | 62 | 35 | 0 |
| LOUISIANA | 3 | 23 | 62 | 56 | 0 |
| MAINE | 2 | 10 | 44 | 17 | 0 |
| MARYLAND | 12 | 40 | 72 | 82 | 10 |
| MASSACHUSETTS | 4 | 18 | 75 | 31 | 6 |
| MICHIGAN | 7 | 121 | 93 | 99 | 5 |
| MINNESOTA | 0 | 16 | 8 | 4 | 0 |
| MISSISSIPPI | 6 | 52 | 97 | 100 | 4 |
| MISSOURI | 3 | 26 | 40 | 22 | 1 |
| MONTANA | 0 | 3 | 3 | 0 | 1 |
| NEBRASKA | 0 | 2 | 0 | 0 | 0 |
| NEVADA | 1 | 9 | 6 | 0 | 0 |
| NEW HAMPSHIRE | 0 | 0 | 2 | 0 | 0 |
| NEW JERSEY | 2 | 72 | 112 | 84 | 4 |
| NEW MEXICO | 3 | 11 | 17 | 8 | 0 |
| NEW YORK | 4 | 49 | 70 | 59 | 4 |
| NORTH CAROLINA | 2 | 8 | 29 | 21 | 3 |
| NORTH DAKOTA | 0 | 8 | 5 | 6 | 0 |
| OHIO | 6 | 32 | 46 | 33 | 4 |
| OKLAHOMA | 0 | 6 | 4 | 1 | 0 |
| OREGON | 4 | 65 | 96 | 32 | 13 |
| PENNSYLVANIA | 7 | 82 | 142 | 73 | 20 |
| PHILIPPINES | 0 | 1 | 0 | 0 | 0 |
| PUERTO RICO | 0 | 15 | 27 | 3 | 1 |
| RHODE ISLAND | 0 | 1 | 1 | 0 | 0 |
| SOUTH CAROLINA | 6 | 62 | 91 | 89 | 12 |
| SOUTH DAKOTA | 0 | 2 | 0 | 0 | 0 |
| TENNESSEE | 3 | 34 | 112 | 30 | 2 |
| TEXAS | 9 | 46 | 35 | 35 | 6 |
| UTAH | 0 | 1 | 2 | 0 | 0 |
| VERMONT | 0 | 0 | 1 | 0 | 0 |
| VIRGINIA | 13 | 73 | 125 | 90 | 15 |
| WASHINGTON | 1 | 20 | 10 | 4 | 1 |
| WEST VIRGINIA | 8 | 59 | 102 | 68 | 14 |
| WISCONSIN | 0 | 9 | 3 | 4 | 1 |
| WYOMING | 0 | 0 | 0 | 1 | 0 |

**Supplementary Table 8. University of Chicago cohort demographics**

|  | | Total (n=3,236) | LIVE 3 (n=731) | LIVE 2 (n=1,074) | LIVE 5 (n=780) | LIVE 6 (n=517) | LIVE 1 (n=134) |
| --- | --- | --- | --- | --- | --- | --- | --- |
| Age (years) (median,IQR) | | 65 (58, 73) | 68 (60, 76) | 65 (57, 73) | 63 (56, 72) | 65 (58, 72) | 68 (60, 78) |
| Female (n,%) | | 1,680 (52%) | 328 (45%) | 640 (60%) | 365 (47%) | 260 (50%) | 87 (65%) |
| Race (n,%) | Black | 2,041 (63%) | 490 (67%) | 658 (61%) | 489 (63%) | 319 (62%) | 85 (63%) |
|  | White | 1,069 (33%) | 213 (29%) | 376 (35%) | 261 (33%) | 178 (34%) | 41 (31%) |
|  | Other | 126 (4%) | 28 (3.8%) | 40 (3.7%) | 30 (3.9%) | 20 (3.9%) | 8 (6.0%) |

**Supplementary Table 9. Survival Statistics of time to death by LIVE Score for University of Chicago cohort**

**
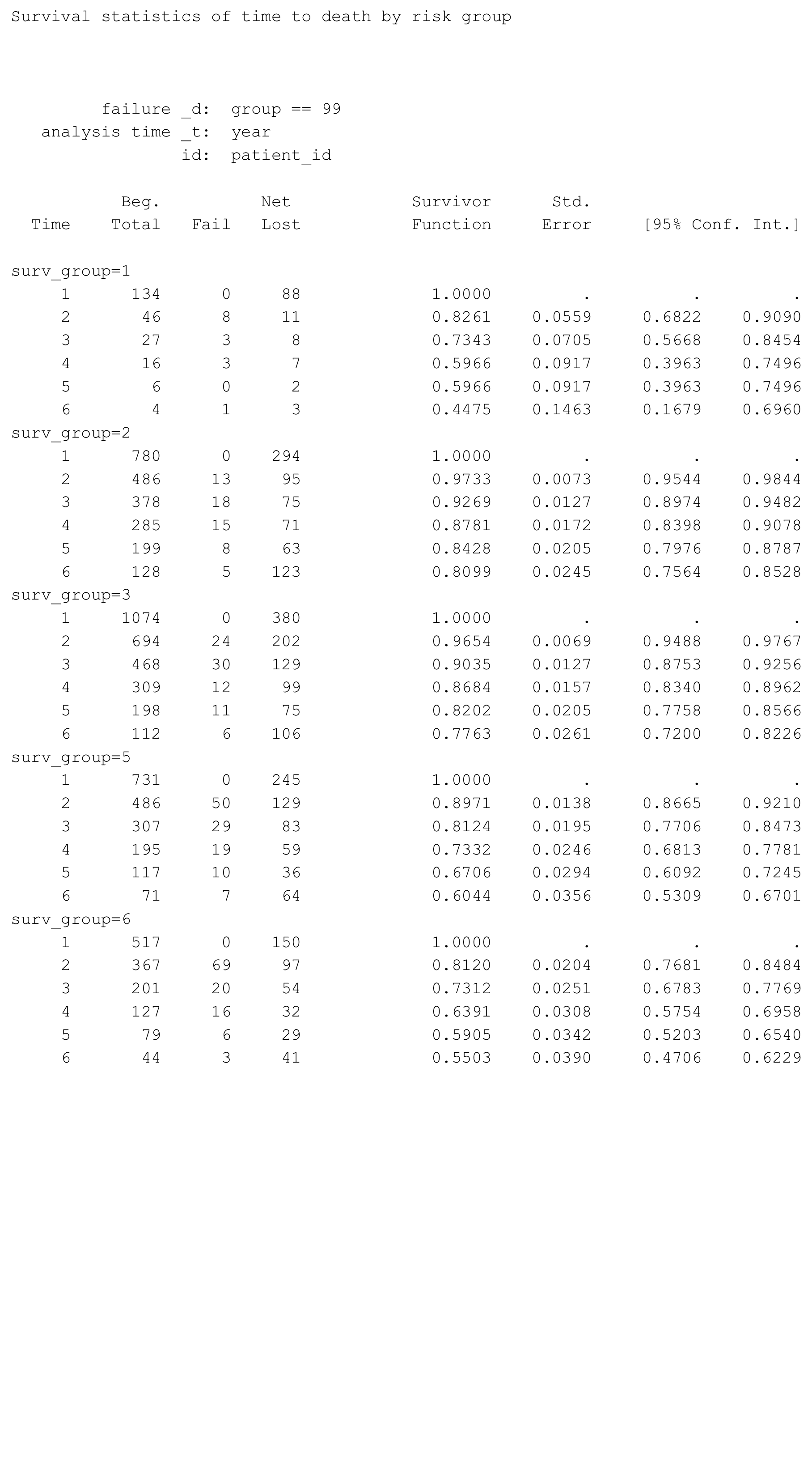
**

**Supplementary Figure 15. Kaplan-Meier Survival for COPD exacerbation for University of Chicago Cohort. Differences among groups are not significant.**

**Supplementary Table 10. Survival Statistics of Time to COPD exacerbation by LIVE Score**

**
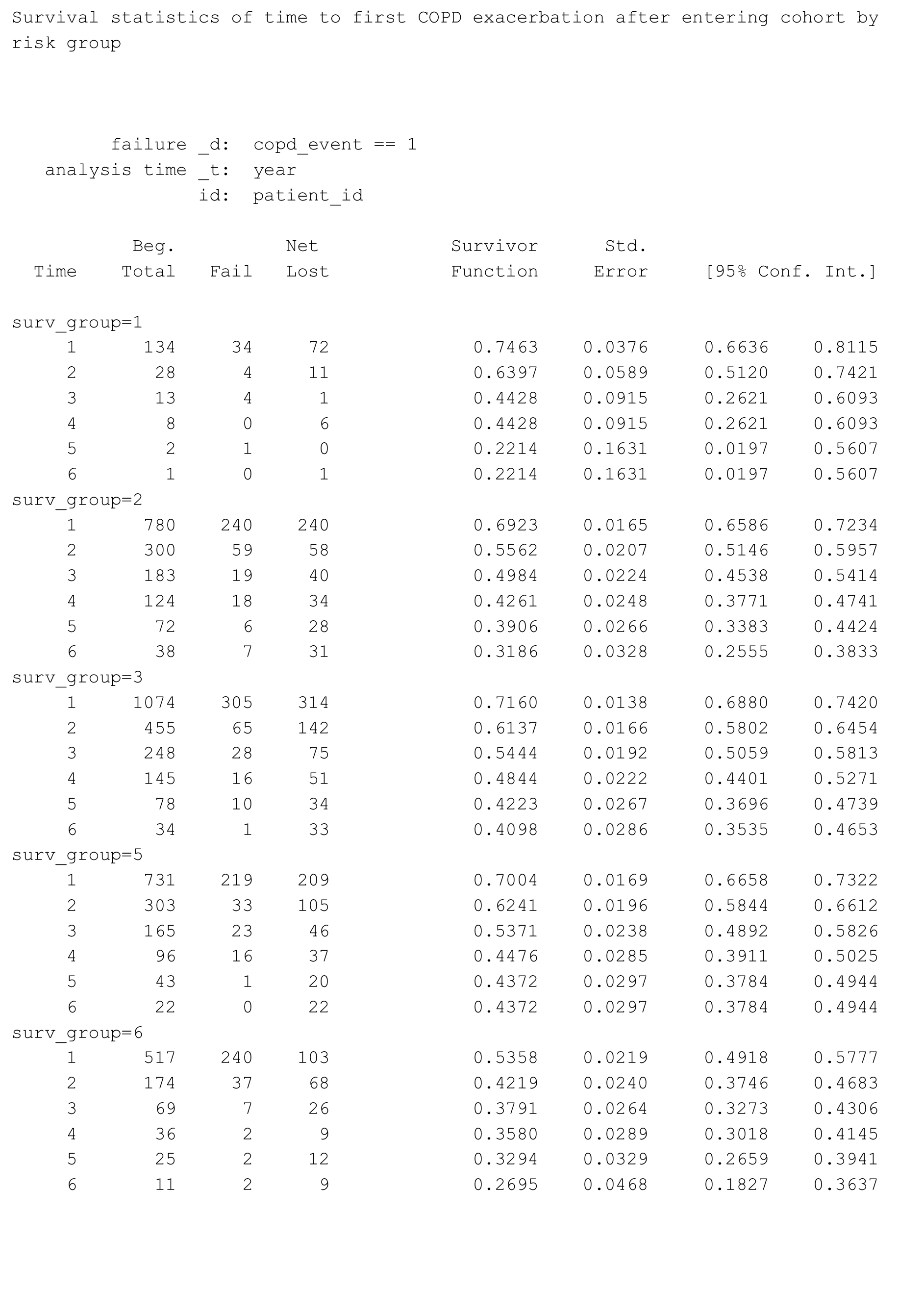
**
